# CuO nanoparticles for green synthesis of significant anti-*Helicobacter pylori* compounds with in silico studies

**DOI:** 10.1038/s41598-024-51708-1

**Published:** 2024-01-18

**Authors:** Wesam S. Shehab, Doaa A. Elsayed, Atef M. Abdel Hamid, Mohamed G. Assy, Samar M. Mouneir, Eman O. Hamed, Sahar M. Mousa, Gehan T. El-Bassyouni

**Affiliations:** 1 Department of Chemistry, Faculty of Science, Zagazig University, Zagazig, 44519 Egypt; 2 Department of Pharmacology, Faculty of Veterinary Medicine, Cairo University, Cairo, 12211 Egypt; 3 Inorganic Chemistry Department, National Research Centre, 33 El-Buhouth St., Dokki, Cairo, 12622 Egypt; 4 Ceramics and Building Materials Department, National Research Centre, 33 El-Buhouth St., Dokki, Cairo, 12622 Egypt

**Keywords:** Biological techniques, Biotechnology, Computational biology and bioinformatics, Drug discovery, Chemistry, Nanoscience and technology

## Abstract

*Helicobacter pylori (H. pylori)* is a universal health intimidation as mentioned by the World Health Organization. The primary causal agent linked to a number of illnesses, including inflammation and the development of stomach ulcers, is Helicobacter pylori. Since, *H. pylori* develops antibiotic resistance quickly, current *H. pylori* treatment approaches are becoming less effective. Our research aims to highlight novel formulation antibiotics using CuO-NPs as catalysts and studied their activity as *anti-helicobacter pylori* supported by computational studies (POM analysis and molecular docking) software. They were designed for *anti-Helicobacter Pylori* action. All compounds revealed a bactericidal effect better than the reference McFarland standards.

## Introduction

Gram-negative, spiral-shaped, microaerophilic *Helicobacter pylori (H. pylori)* bacterium. *H. pylori* infection affects over 50% of people worldwide, with infection rates being greater in underdeveloped nations. The pathophysiology of chronic gastritis, peptic stomach ulcers, gastric mucosa-related lymphomas, and even gastric cancer are all linked to *H. pylori* infection. *H. pylori* was classified by the World Health Organization as a Group I human carcinogen in 1994. *H. pylori* infection causes more than 85% of cases of stomach cancer. There is general agreement that eliminating *H. pylori* can lower the prevalence of stomach cancer^1^. In 1980, the H. pylori genome was fully sequenced after it was obtained from a gastritis patient. With 1, 667, 867 base pairs on its circular chromosome, it is projected to have 1590 coding sequences. The findings imply that the fundamental processes of cell division, secretion, and replication are comparable to those of E. Coli and H. influenza. However, the number of genes found in isolates, or the size of its core genome, varies based on the number of strains examined and where in the world they are from. Furthermore, the capacity of H. pylori species to generate an active vacuolating cytotoxin (VacA) and a cytotoxin-associated protein (CagA) has been linked to their virulence, according to genome analysis. VacA is known to damage epithelial cells, disrupts tight junctions, and causes apoptosis, while CagA may cause inflammation and is a potentially carcinogen agent^2^.

In fact, because *H. pylori* strains differ from one another, the particular strain that affects a person might determine their prognosis. Conversely, during the past several decades, there has been a significant shift in the clinical features and epidemiology of *H. pylori* infections, particularly in poorer nations. In the context of the worldwide growth in antibiotic use in the general population, there is presently a visible failure, mostly owing to *H. pylori* resistance to various antibiotics, despite the use of therapeutic regimens and international standards created by microbiology specialists^3^. Our research aims to highlight novel formulation designs and chemicals to battle the rising rates of antibiotic-resistant *H. pylori* strains, as well as the associated risk factors and global prevalence. To achieve our goal, cyclization of azo compounds to create new families of triazine and diazine derivatives, exhibiting various pharmaceutical activities. Triazine derivatives revealed many activities as antimicrobial^4^, anti-inflammatory^5^, anti-cancer^6^, anti-depressant^7^, anti-bacterial^8^, anti-fungal^9^, antioxidant^10^, antimalarial^11^, anti-proliferative^12^, and analgesic activities^13^. Moreover, diazine derivatives disclosed a wide-ranging of applications^14^. More than half of all the chemicals used in the majority of chemical reactions are produced by catalysis^15^. Numerous industrialized operations, such as oil refining, organic synthesis, and pollution management, now depend heavily on heterogeneous catalysts^16^. It is expected that changing the support through ideologies like nanoscience and nanotechnology or keeping an eye on the pore structure would lead to the development of heterogeneous catalytic activity^17^. The issue of catalyst separation and recovery from the reaction matrix is addressed for heterogeneous catalysis by utilizing a variety of catalyst supports to constrain the particle. This therefore provides a big, suitable surface^18^. The heterogeneous catalyst needs a space so it won't dissolve into the solution matrix^19^. One of this catalyst is nano-CuO which used in this study.

An effective bioinformatic method known as PETRA/OSIRIS/MOLINSPIRATION (POM) analysis is used to evaluate the fundamental physical–chemical properties of molecules (represented as structures) and predict properties such as bioactivity, toxicity, and drug-likeness. This method's name is made up of the programs PETRA, OSIRIS, and the MOLINSPIRATION free online application. A software package called PETRA (Parameter Estimation for the Treatment of Reactivity Applications) consists of numerous empirical techniques for computing fundamental physicochemical parameters of organic compounds. The study team of Prof. J. Gasteiger has created all of the methodologies over the past 20 years, and they are all empirical in character. The heat of formation, bond dissociation energies, sigma charge distribution, π -charge distribution, inductive effect, resonance effect, delocalization energy, and polarizability effect may all be measured with this program^20^.

In the current reportnew composites of triazine and diazine derivatives were prepared and their activity as anti-*Helicobacter pylori* was studied and supported by computational studies (POM analysis and molecular docking) using MOLINSPIRATION, OSIRIS, ProTox-II and Pred-hERG software and using chemical computing groups of Molecular Operating Environment (MOE 2015) software.

## Results and discussion

### Characterization techniques of the copper oxide nanoparticles

**Figure 1:**
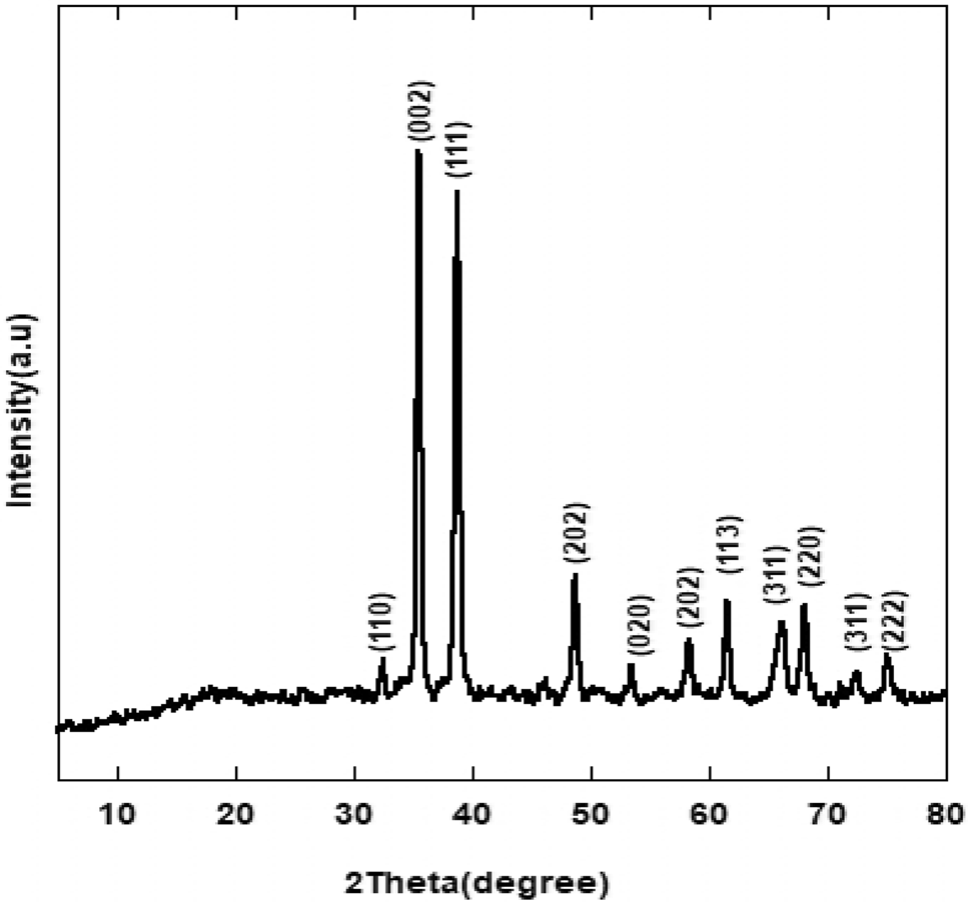
XRD pattern of CuO nanoparticles.

XRD analysis was used to determine the formation of CuO nanoparticles. The crystalline nature of the CuO-NPs and their matching phases were determined using XRD. XRD diffraction peaks at 2θ = 32.35, 35.62, 38.69, 48.72, 53.49, 58.33, 61.57, 66.31, 68.15, 73.12, and 76.15° were assigned in a good agreement to the monoclinic crystallite CuO (JCPDS-05-0661) planes (110), (002), (111), (−202), (020), (202)^21^ as designated in Fig. 1. There were no further peaks attributable to suspected copper hydroxide [Cu(OH)_2_] and/or Cu_2_O, demonstrating the purity of the high grade generated CuO-NPs with monoclinic crystal structure. As a result, it is possible to conclude that the XRD reveals a single phase monoclinic structure of CuO-NPs. The end result was comparable to statistics reported elsewhere^22^.

**Figure 2:**
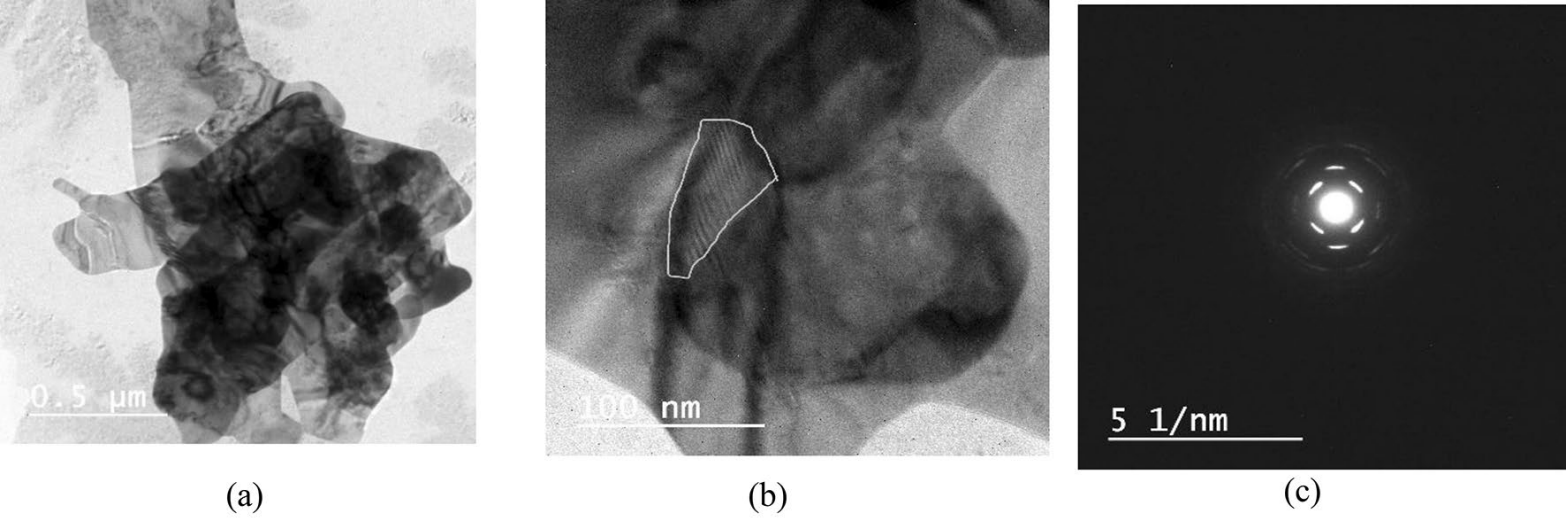
HR-TEM images of CuO-NPs (**a** & **b**) Micrograph image illustrate particles with sheet-like structures, (**c**) Diffraction pattern elucidate the crystallinity of sample.

The HR-TEM image revealed detailed morphological information about the CuO-NPs (Fig. 2a and b). Particles were made up of a sheet-like building structure^23^. The nanoparticles coincided with one other which greatly aided the growth of the flower like nanostructure together with oval and spherical outlines^24^. Furthermore, a crystallographic experimental approach known as "Selected Area (Electron) Diffraction (SAED) pattern enclosed bright circular patches that corroborated the polycrystalline nature, implying that the CuO particles have various crystallographic directions, as seen in Fig. 2c ^23^.

**Figure 3:**
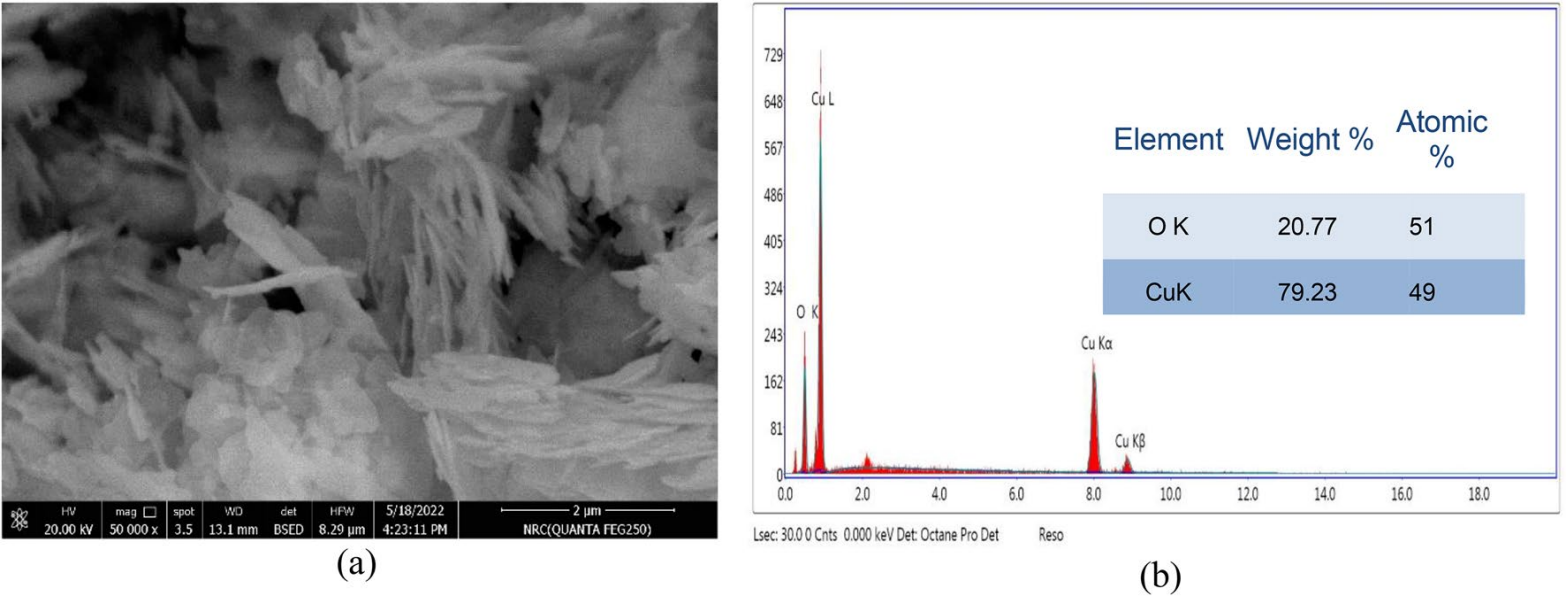
SEM image (**a**) and EDX spectrum (**b**) of CuO nanoparticles.

The structural analysis using SEM is presented in Fig. 3a, which displays the morphological progression of the CuO-NPs, which were discovered to be flakes or plate-like structures similar to the petals of a flower. The attained CuO floweret-nanostructures are talented nominee for possible use in catalysis^25^. The elemental chemical composition of the nanoparticles was investigated by EDX analysis (Fig. 3b). EDX analysis was used to determine the elemental chemical composition of the nanoparticles (Fig. 3b). It found that the chemical makeup was 49% copper and 51% oxygen in atomic percent. This finding confirmed that the CuO-NPs were pure^26^. This outcome was elucidated by microscopic evaluations and spectroscopy^25^.

**Figure 4:**
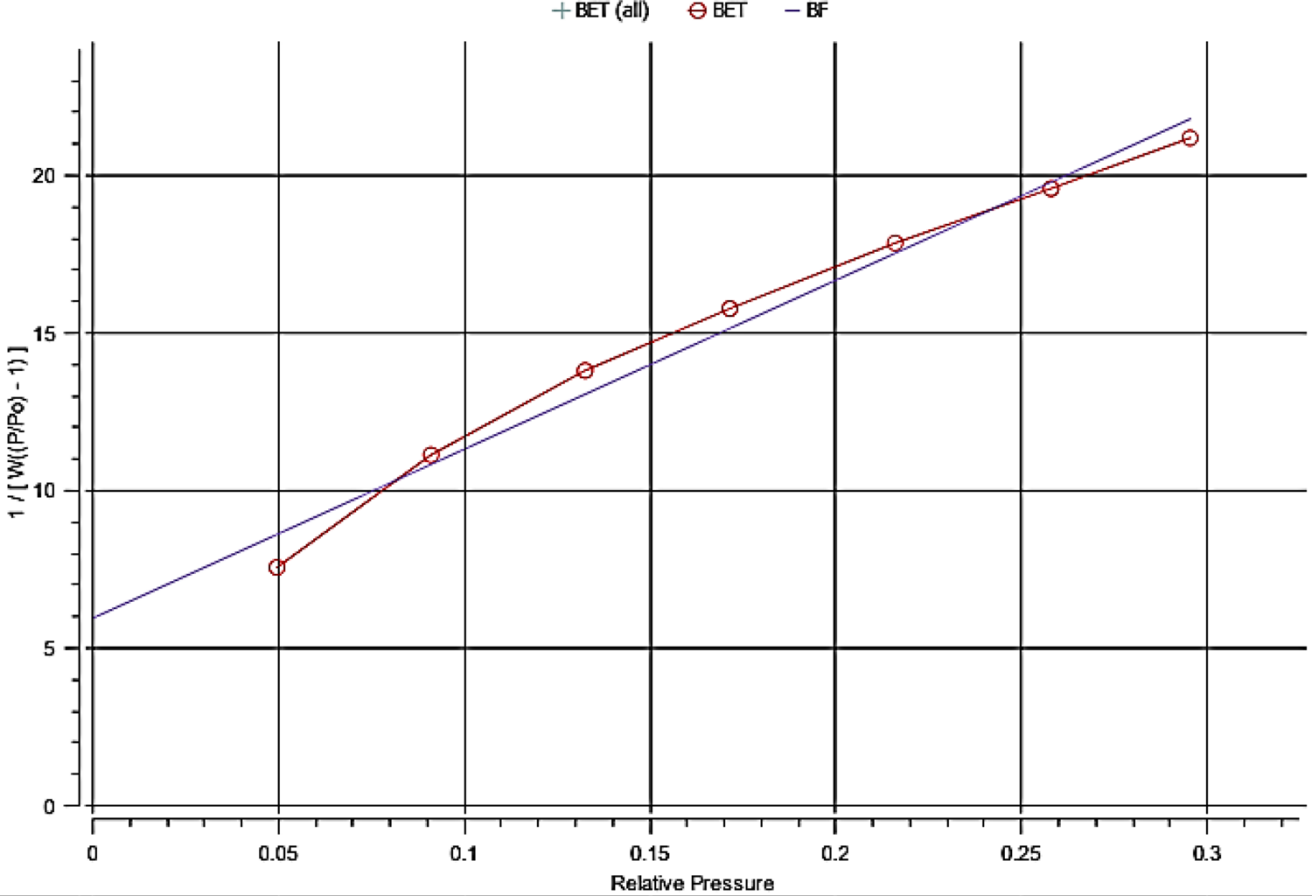
Specific surface area (SSA) of CuO nanoparticles.

The “Specific Surface Area (SSA)” is a distinctive substance that plays an important function in nanoparticles due to the large ratio of surface to volume with decreasing particle size. Adsorption, heterogeneous catalysis, and surface reactions all rely on it. The CuO-NPs' large surface area aided the reaction/interaction between CuO and the interacting media, which occurs often on the surface or at the interface and is influenced by the material's surface area. The surface area of the produced CuO-NPs was 58.4419 (m^2^/gm)was presented in Fig. 4, as sustained by Quantachrome TouchWinTM, model NOVA touch 4LX^27^.

### Synthesis of diazenyl benzoic acid derivatives.

**Figure 5:**
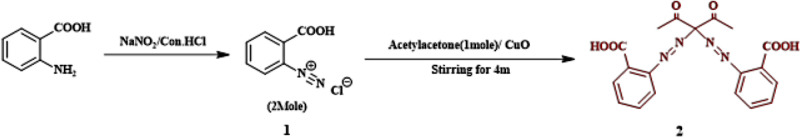
The synthesis of 2,2′-((2,4-dioxopentane-3,3-diyl)bis(diazene-2,1-diyl))dibenzoic acid.

Earlier it was designated that the acetylacetone supplemented with one equivalent of diazonium salt managed to yield 2-[(2,4-dioxopentan-3-yl)diazenyl]benzoic acid or 1-[4-(4-Acetyl-5-methyl-1H-1,2,3-triazol-1-yl)-phenyl]ethenone based on stirring or reflux conditions^28^. While in the present protocol, two amounts of **1** was coupled with acetylacetone in presence of CuO-NPs as a catalytic providing 2,2′-((2,4-dioxopentane-3,3-diyl) bis(diazene-2,1-diyl)) dibenzoic acid **2**^29^ Fig. 5.

To attain the finest kind of catalysts, sodium acetate (C_2_H_3_O_2_Na) and CuO-NPs production were correlated. It became apparent that the finest features were attained when the CuO-NPs was elaborated in the reaction. To amend the time, the model reaction was accomplished under stirring conditions. Organized results verified that 99% yield was reached after 4 min upon consuming of the CuO-NPs as a catalyst (Table 1). The tabulated results showed that CuO-NPs achieved best results in time and yield.

**Table 1:** Optimization of the reaction conditions.

Entry	Catalyst (base)	Condition	Time (m)	Yield (%)
1	Sodium acetate	Stirring	60	97
2	CuO-NPs	Stirring	4	99

The IR spectrum of **2** demonstrated a broadband at 3444.87–3360 cm^−1^, consistent to the OH in the carboxylic group, and the peaks at 1678, 1624and 1450 cm^−1^ were along with (2C=O for carboxylic group), (2C=O for ketones) and (2 N=N), respectively. For the ^1^H-NMR it exhibited *δ at* 15.843 ppm for 2-OH which was replaceable by D_2_O*.* ^13^C-NMR spectra exhibited the carbon of the carbonyl group (C=O) at 197.29 ppm and the carbon of acid at 168.21 ppm*.*MS (*M/Z*): molecular peak was elucidated at (396.56), while base-peak emerged at (207.28), reported in^29^***.***

**Figure 6:**
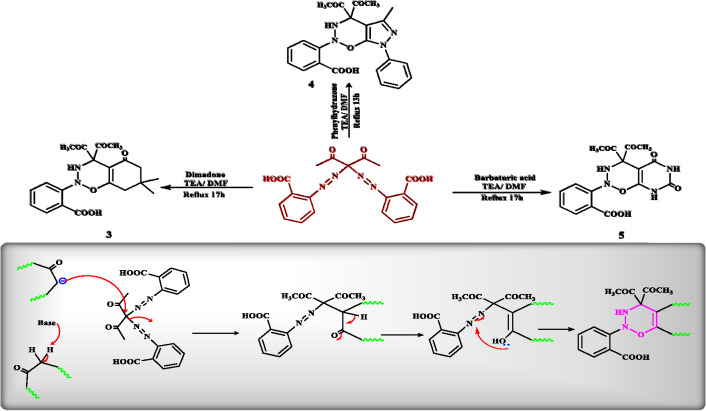
The synthesis of oxadiazin benzoic acid derivatives.

The reaction of 2-((2,4-dioxopentan-3-yl)diazenyl)benzoic acid with different reagents deliberated as a preparatory point for the synthesis of new collections of compounds exhibiting numerous pharmaceutical activities, where heterocyclization of 2-((2,4-dioxopentan-3-yl)diazenyl)benzoic acid **2** by reacting with Dimadone & phenyl hydrazone in DMF in presence of TEA as basic medium provided 2-(4,4-diacetyl-7,7-dimethyl-5-oxo-3,4,5,6,7,8-hexahydro-2*H*-benzo[*e*][1,2,3]oxadiazin-2-yl)benzoic acid **(3)** & 2-(4,4-diacetyl-5-methyl-7-phenyl-4,7-dihydropyrazolo[4,3-*e*][1,2,3]oxadiazin-2(3*H*)-yl)benzoic acid **(4)**, respectively. Their structures were proved by the disintegration of the azo group in IR spectra and the appearance of -NH group as broad band in the IR spectra at 3082 and 3080 cm^−1^ for compounds **3** and **4**, respectively, and as single signal in ^1^H-NMR spectra 7.94 and 7.93 ppm for compounds **3** and **4**, respectively. While the treatment of compound **2** with barbaturic acid in DMF in the incidence of TEA as basic medium gave 2-(4-acetyl-4-(methoxy-l2-methyl)-5,7-dioxo-3,4,5,6,7,8-hexahydro-2*H*-pyrimido[5,4-*e*][1,2,3]oxadiazin-2-yl)benzoic acid **(5)**,The produced compound was proved by IR, ^1^H-NMRspectra and elemental analysis, shown in **scheme (2)**. A promising mechanism for the reaction is presented in Fig. 6.

**Figure 7:**
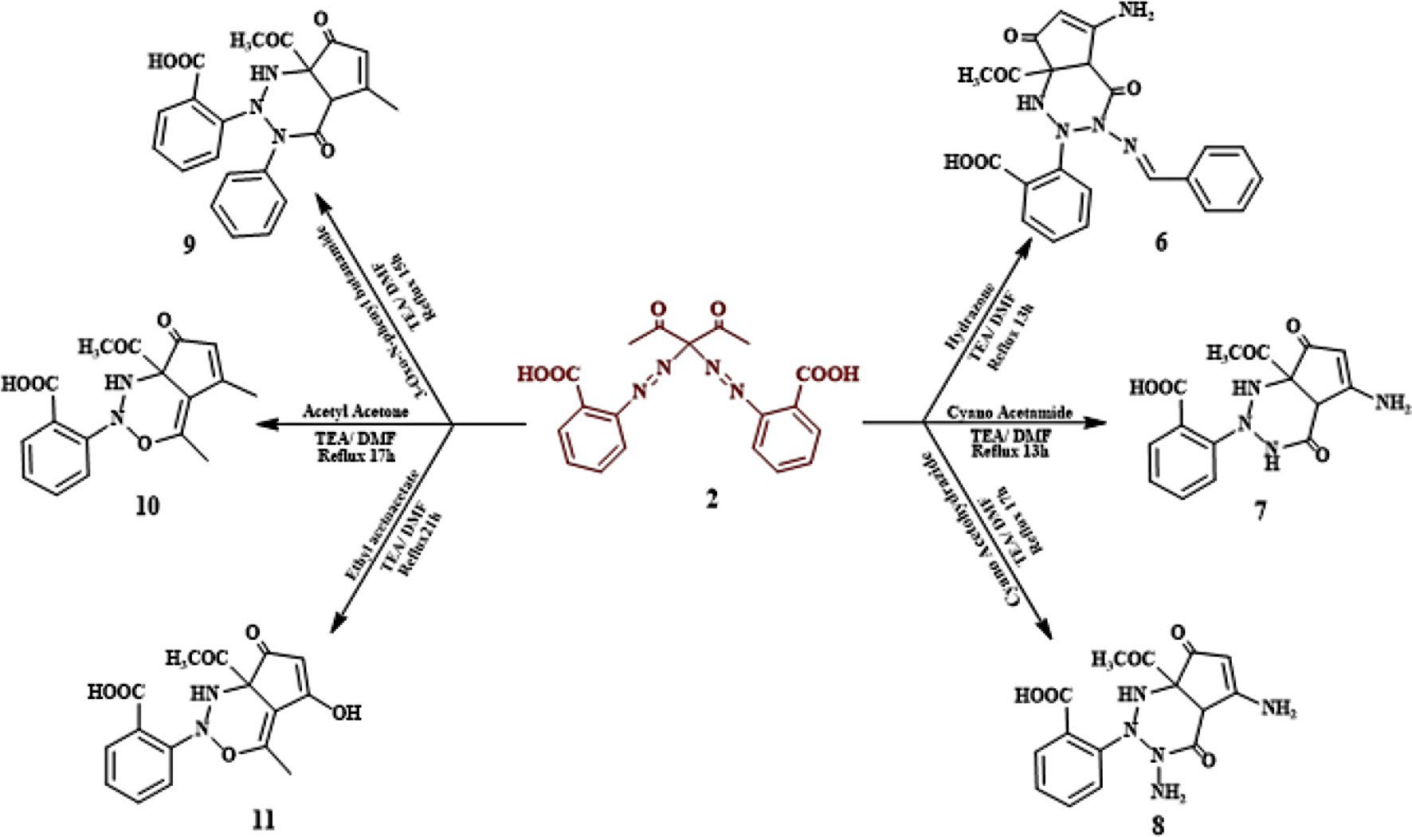
Reaction of azo-compound **2** with different reagents.

**Figure 8:**
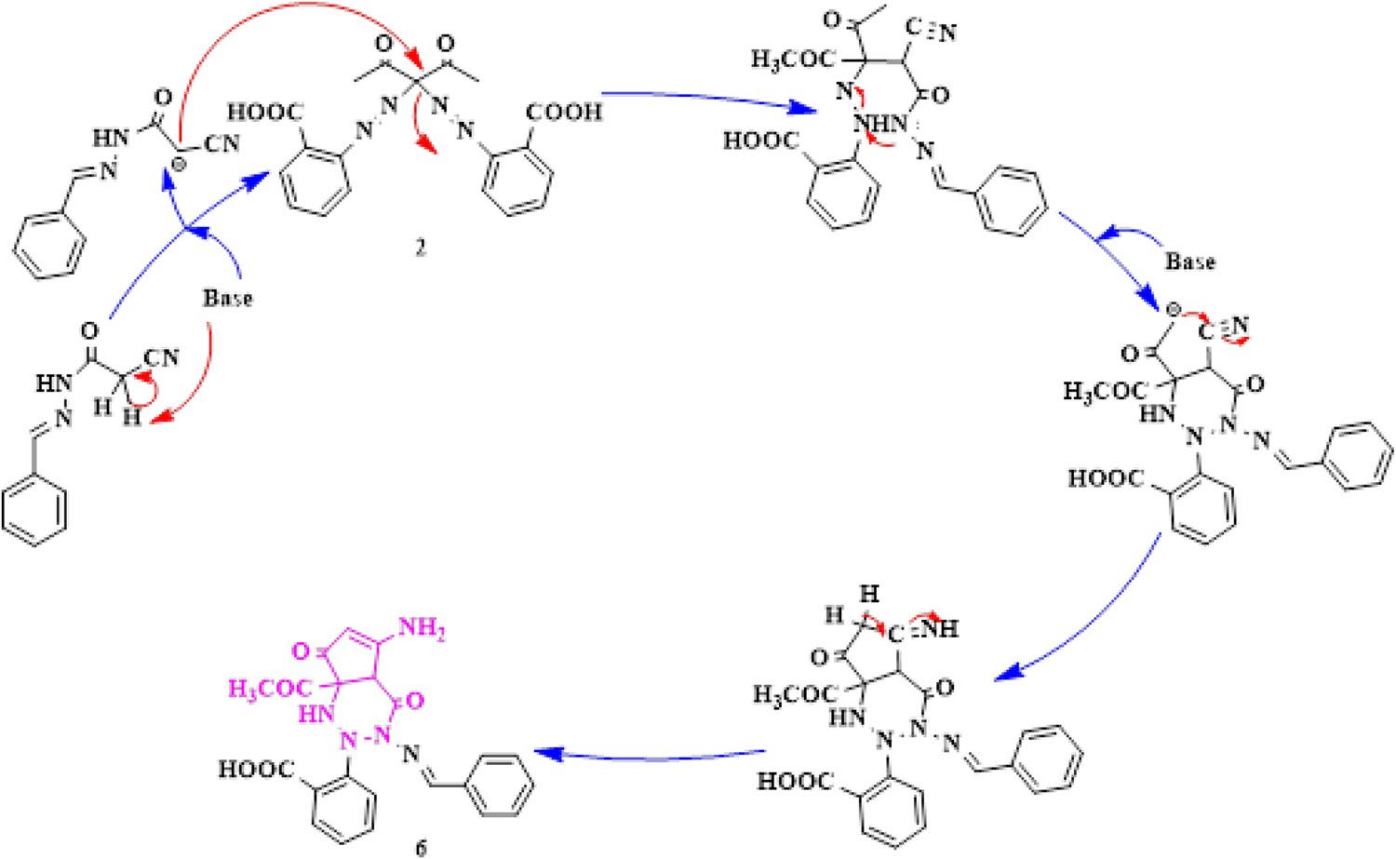
The plausible mechanism for the synthesis of compound **6**.

The reaction of compound **2** with hydrazone, cyanoacetamide and cyano aceto hydrazide under reflux condition in the incidence of TEA resulted in the initiation of compounds **(6, 7 & 8)**, respectively. For compound **6**, IR demonstrated 3426 (OH), 3216(NH), 2927 (CH for aliphatic), 1717 (C=O of ketone), 1702 (C=O of acid), 1674 (C=O of amide), 1600 (C=N), respectively. As well, the ^1^H-NMR spectra showed the appearance of the—3CH groups, 2NH and -NH_2_ signals at *δ* 3.979, 5.387, 7.722, 7.340, 7.722, 7.378 ppm, respectively. Similarly, the structure of compounds **7 & 8** was offered by elemental analysis and spectral data observed in the experimental section Fig. 7. A possible mechanism for the reaction of compound **6** is revealed in Fig. 8. The reaction of compound **2** with 3-Oxo-N-phenyl butanamide, Acetyl Acetone and Ethyl acetoacetate, respectively, directed to the formulation of compounds **(9, 10 & 11)**, whose structure were provided by elemental analysis and spectral data as recognized in the experimental section, presented in Fig. 7.

**Figure 9:**
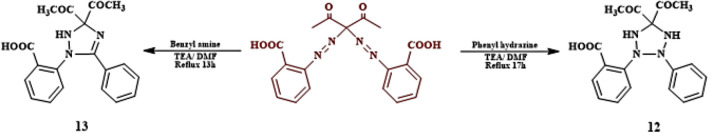
Reaction of compound 2 with phenyl hydrazine and benzyl amine reagents.

**Figure 10:**
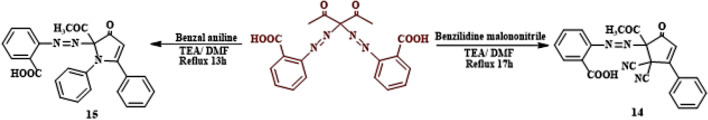
The synthesis of diazenyl benzoic acid derivatives.

In Fig. 9, chemical **2** was refluxed with phenyl hydrazine and benzyl amine in DMF, which was grounded to produce compounds **12** & **13** respectively. Compound **12,** structure was validated by its proper elemental analysis. Its IR spectra revealed broad functional group at 3057 cm^−1^ indicatives for the NH and the appearance of more aromatic ring in the ^1^H-NMR spectra. While compound **13'**s structure was confirmed from its precise elemental analysis, the IR spectra, showed new functional group bands at 3078 cm^-1^, broad one for NH and at 1600 cm^−1^ for the cyano group (C=N), and the appearance of more aromatic ring on ^1^H-NMR spectra with vanishing of the azo group. When compound **2** reacted with benzilidine malononitrile and benzal aniline in refluxing DMF containing few drops of TEA, it provided desired compounds of **14 & 15**, respectively as presented in Fig. 10. The structure of compounds **14 & 15** was provided by elemental analysis and spectral data as realized in the experimental section.

### Anti-H. Pylori activity

#### Determination of inhibitions zones of samples

**Figure 11:**
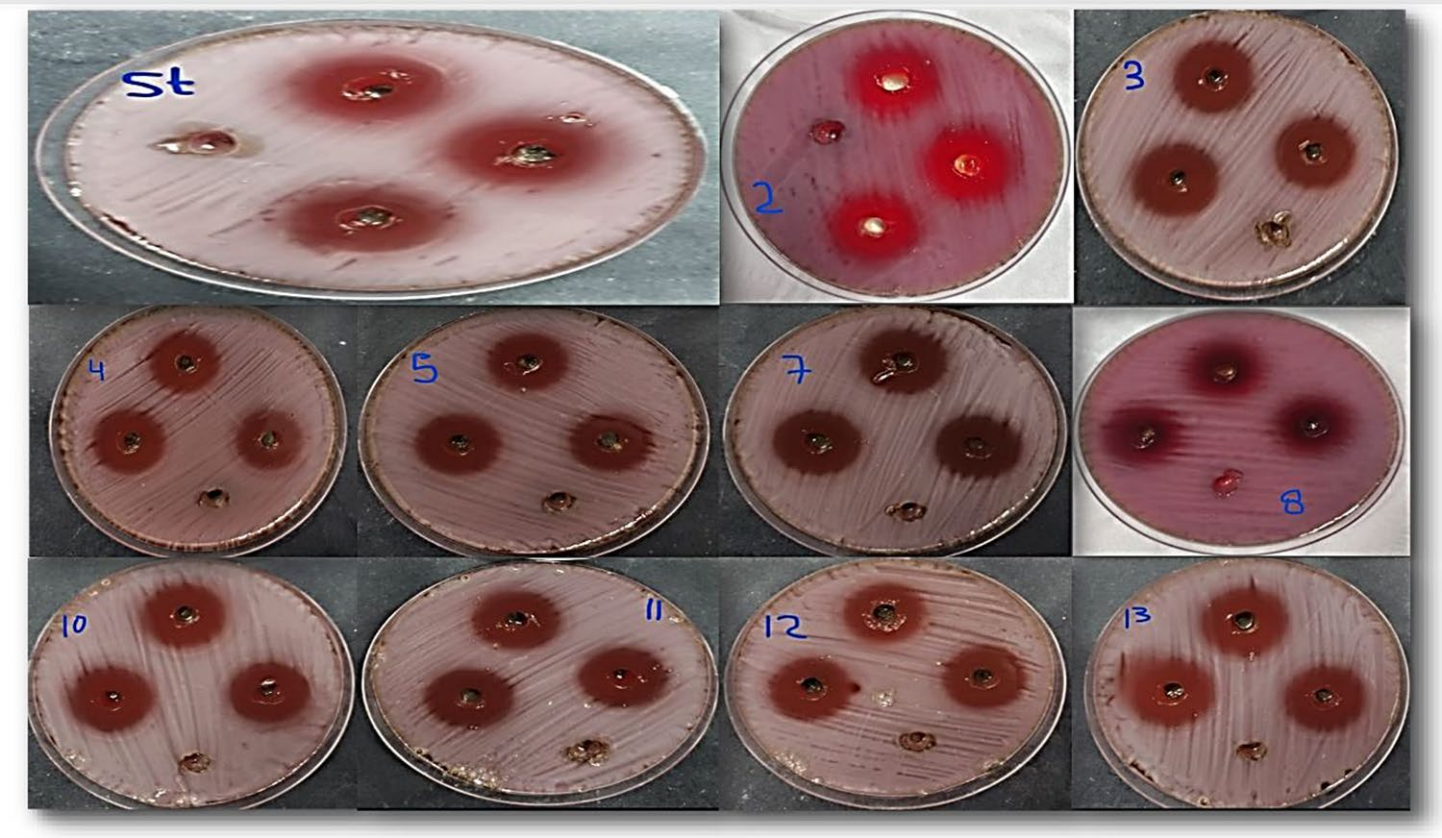
The antibacterial effect of synthesized compounds against *H. Pylori.*

Results revealed that the inhibitions zones in millimeters of samples **2, 3, 4, 5, 7, 8, 10, 11, 12** and **13** on the *Anti-H. Pylori* were 22.33, 21.00, 19.67, 18.33, 23.67, 17.67, 22.00, 21.33, 23.33, and 25.00 mm, respectively as indicated in (Table 2). All compounds have bactericidal effect better than the reference McFarland standards (used to modify the turbidity of bacterial suspensions so as to the number of bacteria will be within a given range to homogenize microbial testing) which has an inhibition zone of 19.67 mm Fig. 11. DEMSO was used as a control negative as it was used for dissolving the samples and it had not any antibacterial activity. The data were analyzed using IBM SPSS Statistics (Version 27)^30^**.**

**Table 2:** Determination of compounds antibacterial activity on Pathogenic *H. pylori*.

Sample Code	Inhibition zone(mm)
2	22.33 ± 0.33bcd
3	21.00 ± 0.00de
4	19.67 ± 0.33ef
5	18.33 ± 0.33f.
7	23.67 ± 0.33ab
8	17.67 ± 0.33f.
10	22.00 ± 0.58bcd
11	21.33 ± 0.88cde
12	23.33 ± 0.33abc
13	25.00 ± 0.58a
Stander (clarithromycin, amoxicillin, metronidazole)	19.67 ± 0.33ef
Control negative (DEMSO)	N.D

Data represented as mean of 3 inhibition zones ± SEM—Standard error of the mean, the column with different superscripts were statistically significant at *p* value ≤ .05 (One Way Analysis of Variance (ANOVA), Tukey's post-hoc test).

*N.D* Not Detected.

The anti-Helicobacter activity of newly synthesized compounds was assessed in relation to previous studies, Rüegg et al.^31^ corroborated the discovery of a novel derivative, 3-farnesyl-2-hydroxy benzoic acid, in Piper multiplinervium leaves. This compound exhibited substantial anti-*Helicobacter pylori* activity at 37.5 µg/ml and demonstrated efficacy against various bacteria and fungi, including Staphylococcus aureus, Escherichia coli, Klebsiella pneumoniae, Mycobacterium smegmatis, Pseudomonas aeruginosa, and Candida albicans, with MICs ranging from 2.5 to 5 µg/ml. Its structure was elucidated using MS, ^1^H, and ^13^C NMR techniques. These findings support the potential use of benzoic acid derivatives in addressing stomach discomfort, aligning with observed anti-*Helicobacter pylori* activity. Compound 12 emerged as the safest option for anti-Helicobacter use, exhibiting a predicted LD_50_ of 8000 mg/kg^32^ and a toxicity class of six, as determined by the Protox II virtual lab for molecular toxicity analysis in a rat model. Additionally, Compound 12 displayed significant anti-Helicobacter activity, surpassing standard drugs with a zone of inhibition of 23.33 mm, MIC of 3.9 µg/ml, and MBC of 7.8 µg/ml compared to 19.67 mm, 1.95 µg/ml, and 1.95 µg/ml for the standard drug, respectively.

#### Minimal inhibitory concentration (MIC) and minimal bactericidal concentration (MBC)

Table 3 elucidates that all the tested samples have bactericidal effect where MBC/MIC Index for all tested samples was ≤ **4.** The index of MBC/MIC of samples 3, 8 & 12 was two folds that of other samples.

**Table 3:** Determination of samples concentration (MIC) and (MBC).

Sample code	MIC (µg/mL)	MBC (µg/mL)	MBC/MIC index
2	31.25	31.25	1
3	15.6	31.25	2
4	31.25	31.25	1
5	62.5	62.5	1
7	7.8	7.8	1
8	125	250	2
10	15.6	15.6	1
11	15.6	15.6	1
12	3.9	7.8	2
13	1.95	1.95	1
Stander	31.125	62.5	2

**NB:** If the MBC/MIC index of the samples ≤ 4 suggested their bactericidal activity against *H. pylori*, while the MBC/MIC index of the samples > 4 demonstrated their bacteriostatic activity.

### In silico studies

#### POM analysis

POM analysis and similar methods are important tools for determining different physico-chemical characteristics and forecasting a molecule's biological activity, ADME parameters, and toxicity. Compounds **7**, **12**, and **13** were subjected to a modified POM analysis utilizing the MOLINSPIRATION, SWISSADME, and OSIRIS tools.

##### OSIRIS calculation

To predict physico-chemical (MW, clogP, water solubility, and total polar surface area-TPSA) and toxicological (mutagenic effect, tumorigenicity, irritancy, and reproductive toxicity) molecular properties, Thomas Sander created the free program OSIRIS (full name Osiris Property Explorer). The software determines drug score and drug-likeness qualities based on these factors^21^.

**Figure 12:**
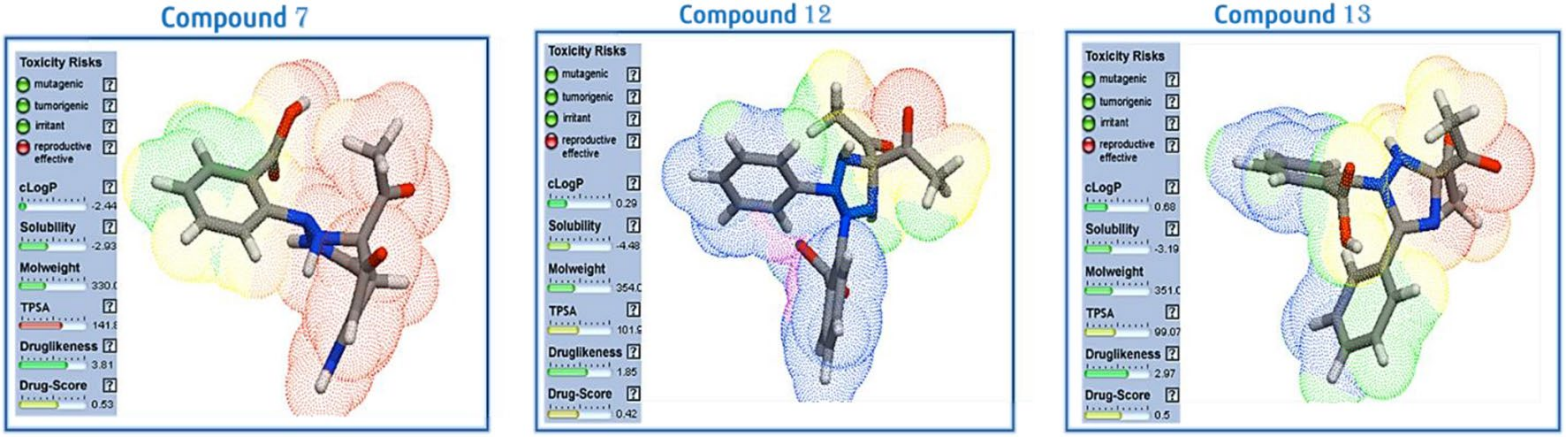
OSIRIS Calculation of molecular properties of compounds 7, 12 and 13.

The expected toxicity dangers for chemicals 7, 12, and 13 were calculated using the OSIRIS tool Fig. 12, and the results are shown in Fig. 12. According to normal clinical medications, these compounds produce less adverse effects. Additionally, it was shown that substances 7, 12, and 13 have some pharmacomodulation and may behave as antibiotics (DS = 0.53, 0.42, and 0.5, respectively) where toxicological for these compounds have some reproductive toxicity and have TPSA more than 140 Å^2^where (TPSA score ideal for drug-like molecules is less than 140 Å^2^).

##### Molinsipration calculation

A wide variety of computational biology programs are available through MOLINSPIRATION that help with the manipulation and processing of molecules. These tools include SMILES and Sdfile conversion, molecule normalization, tautomer generation, molecule fragmentation, calculation of various molecular properties required for QSAR, molecular modeling and drug design, high-quality molecule depiction, and molecular database instruments assisting substructure and likeness searches^22, 33^.

Table 4 lists the anticipated pharmacokinetic/Molinspiration parameters for the chemicals produced **7**, **12**, and **13**. With the use of Molinspiration online screening, almost all of the compounds produced had potential biological activity, as demonstrated by the docking parameters in Table 5, which highlight the drug-like properties against kinase inhibitors, protease, and enzyme inhibitors. The Calculated distribution of activity scores (version 2022.08) are compared to scores for GPCR ligands, kinase inhibitors, ion channel modulators, nuclear receptor ligands, protease inhibitors, and other enzyme targets. These scores contain scores for over 100,000 common druglike compounds. Effective differentiation between active and inactive molecules is made possible by the score.

**Table 4:** Physicochemical properties of the synthesized compounds.

Compound	miLogP	TPSA	n-atoms	M.W	nON	nOHNH	n-violations	n-rotb	Volume
7	− 0.17	141.83	24	330.30	9	5	0	3	272.50
12	2.30	101.97	26	354.37	8	3	0	5	309.58
13	1.69	99.07	26	351.36	7	2	0	5	307.65

**Table 5:** Physicochemical Molinspiration bioactivity score.

Compound	GPCR ligand	Ion channel modulator	Kinase inhibitor	Nuclear receptor ligand	Protease inhibitor	Enzyme inhibitor
7	− 0.05	− 0.28	− 0.38	− 0.13	− 0.05	− 0.02
12	− 0.12	− 0.22	− 0.34	− 0.07	− 0.09	0.02
13	− 0.20	− 0.31	− 0.43	− 0.24	− 0.06	− 0.09

#### ProTox-II and Pred-hERG

**Figure 13:**
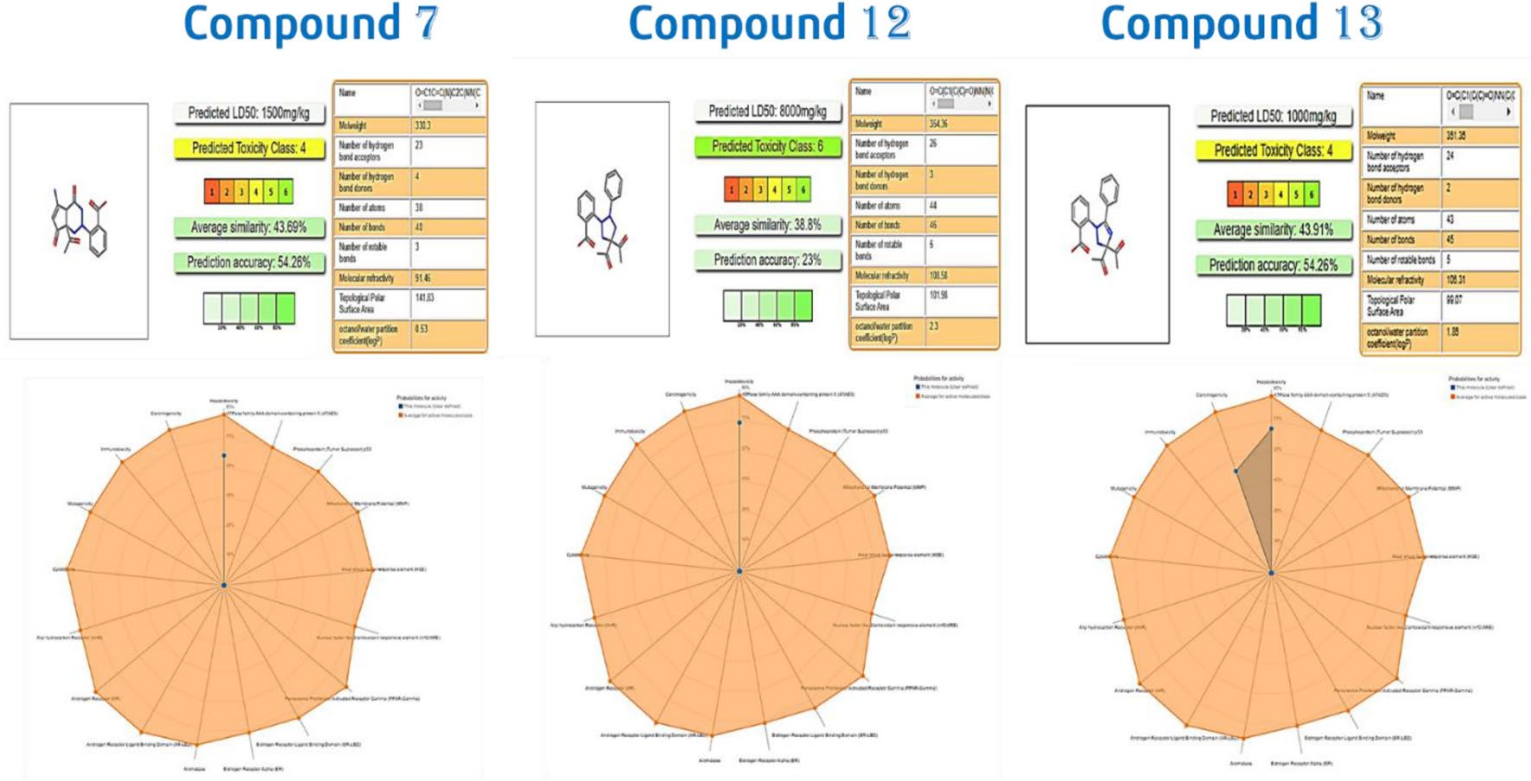
The oral toxicity prediction results &the toxicity radar chart is intended to quickly illustrate the confidence of positive toxicity results compared to the average of its class for compounds 7, 12 and13.

Protox II virtual lab for the analysis of little molecule toxicity. Identifying chemical toxicity is a crucial step in the creation of new pharmaceuticals. According to the ProTox-II, the oral LD50 values for the three chemicals in a rat model range from 159 to 2480 mg/kg, with quercetin having the lowest value and (1 s, 4 s)-Eucalyptol having the highest. Figure 13 displays the comparison of chemicals **7**, **12**, and **13** to those in the dataset where Predicted toxicity class for compound 12 where 6 which have high Predicted LD50 (mg/kg).

##### Pred-hERG

**Figure 14:**
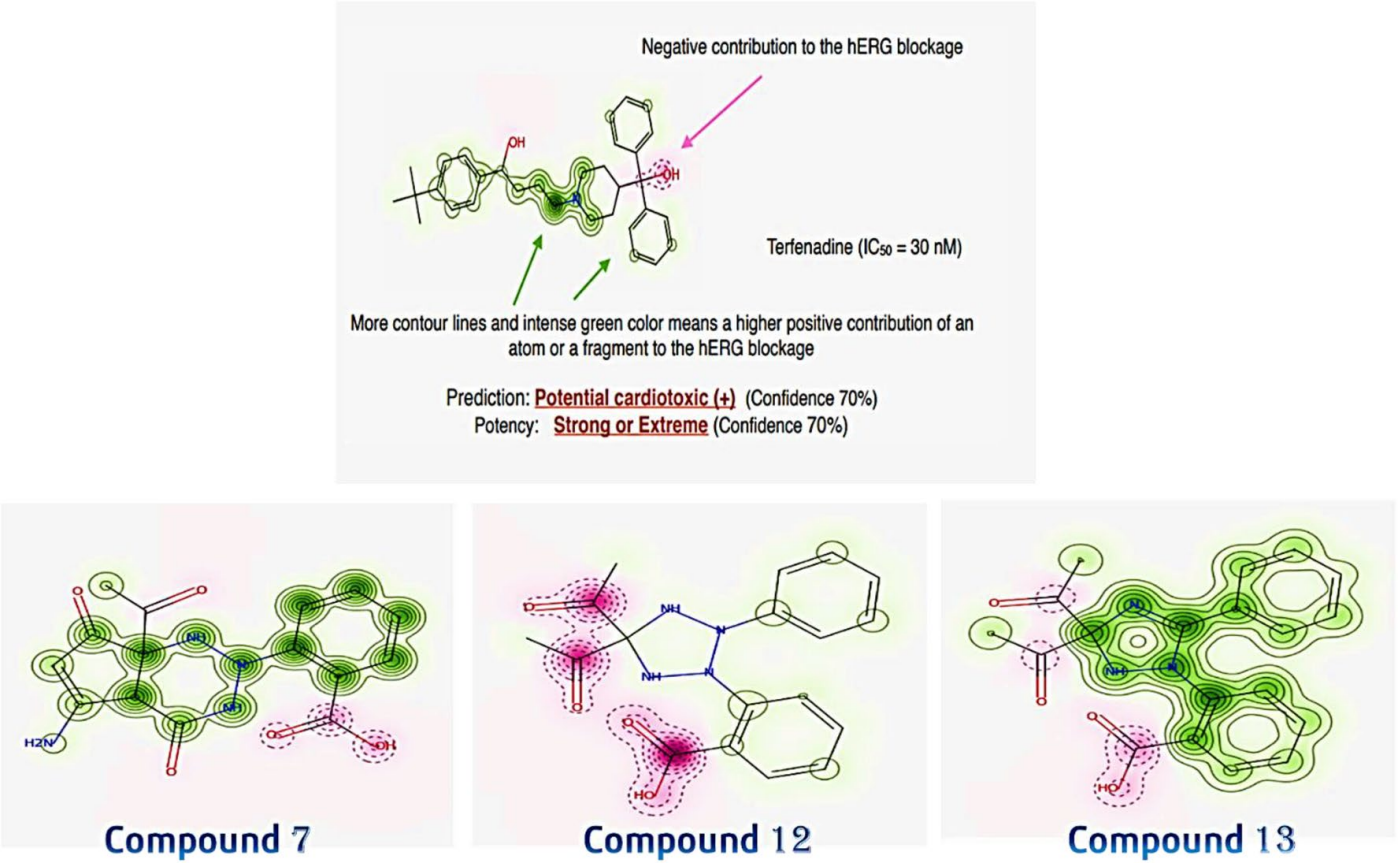
Pred-hERG Results Probability Map of compounds 7, 12 and13.

Biologically varied protein targets are frequently bound by chemically related substances, although protein structures may not always recognize the same ligands. By interpolating the output prediction equalized by the compound similarity criteria, pharmacological and off-target connections between proteins and a ligand set assist increase the machine learning confidence. This pipeline contributes to lowering the false-negative error and improving forecasts of off-target medication effects. One of the key ideas in cheminformatics is chemical similarity. The 2D Tanimoto technique utilized here is one that is frequently used to determine these similarity algorithm metrics. The final Tanimoto coefficient is fingerprint-based, encoding each molecule to a fingerprint “bit” location (MACCS), with each bit recording whether a molecule fragment is present (“1”) or not (“0”) in the sample. The potency results are represented in Table 6b, While Fig. 14 shows the Probability Map of compounds **7, 12**, **13**.

**Table 6:** The predicted toxicity for 7, 12 and 13 using: (a) ProTox-II and (b) Pred-hERG software.

	7	12	13
a. Pro-ToxII			
Predicted LD50 (mg/kg)	1500mg/kg	8000mg/kg	1000mg/kg
Predicted toxicity class	4	6	4
Average similarity (%)	43.69%	38.80%	43.91
Prediction accuracy (%)	54.26%	23.00%	54.26
b. Pred-hERG			
Prediction/potency	Non-cardiotoxic (−)	Non-cardiotoxic (−)	Non-cardiotoxic (−)
Confidence (%)	70	70	70
Applicability domain (A.D.)	Yes (Value = 0.26 and limit = 0.26)	Yes (Value = 0.26 and limit = 0.26)	Yes (Value = 0.26 and limit = 0.26)

#### Molecular docking study

Molecular docking analysis was directed to study the contact of the natural compounds with several molecular targets of anti-inflammatory activity. Molecular docking analysis for the generated database was used to investigate the hypothesized mechanism of achievement for the newly developed and produced drug for bactericidal activity against *H. pylori* compared to a standard reference (4R,6R,7S)-2-(2-cyclopropylethyl)-4,6,7-trihydroxy-4,5,6,7-tetrahydro-1-benzothiophene-4-carboxylic acid [JPS] (PDB code: 2XDA)^34^. Such analysis was carried out to obtain further insight into the binding modes of the synthesized compounds into the protein-binding site of type II dehydroquinase enzyme.

**Figure 15:**
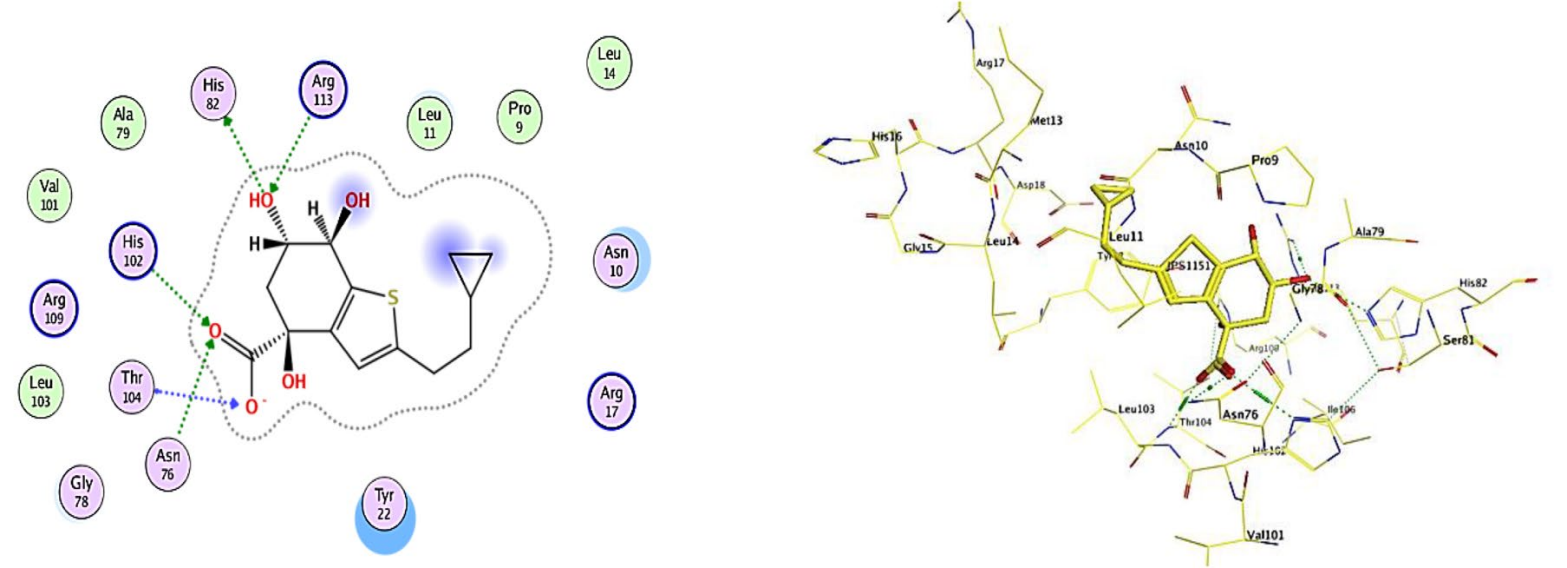
2D receptor interactions and 3D receptor interactions of the ligand **JPS** as a reference for bactericidal activity against *H. Pylori.*

To confirm the current docking investigation at the active site, the co-crystallized ligand JPS was re-docked utilizing a similar collection of parameters. The best-docked pose's root mean square deviation (RMSD) was 2.1414 Å and its energy score was − 7.36 (Kcal/mol), supporting the MOE software's docking research. As seen in Fig. 15 JPS created an ionic bond with His102 and six hydrogen bonds with His82, Thr104, Asn76, His102, and Arg113.

**Figure 16:**
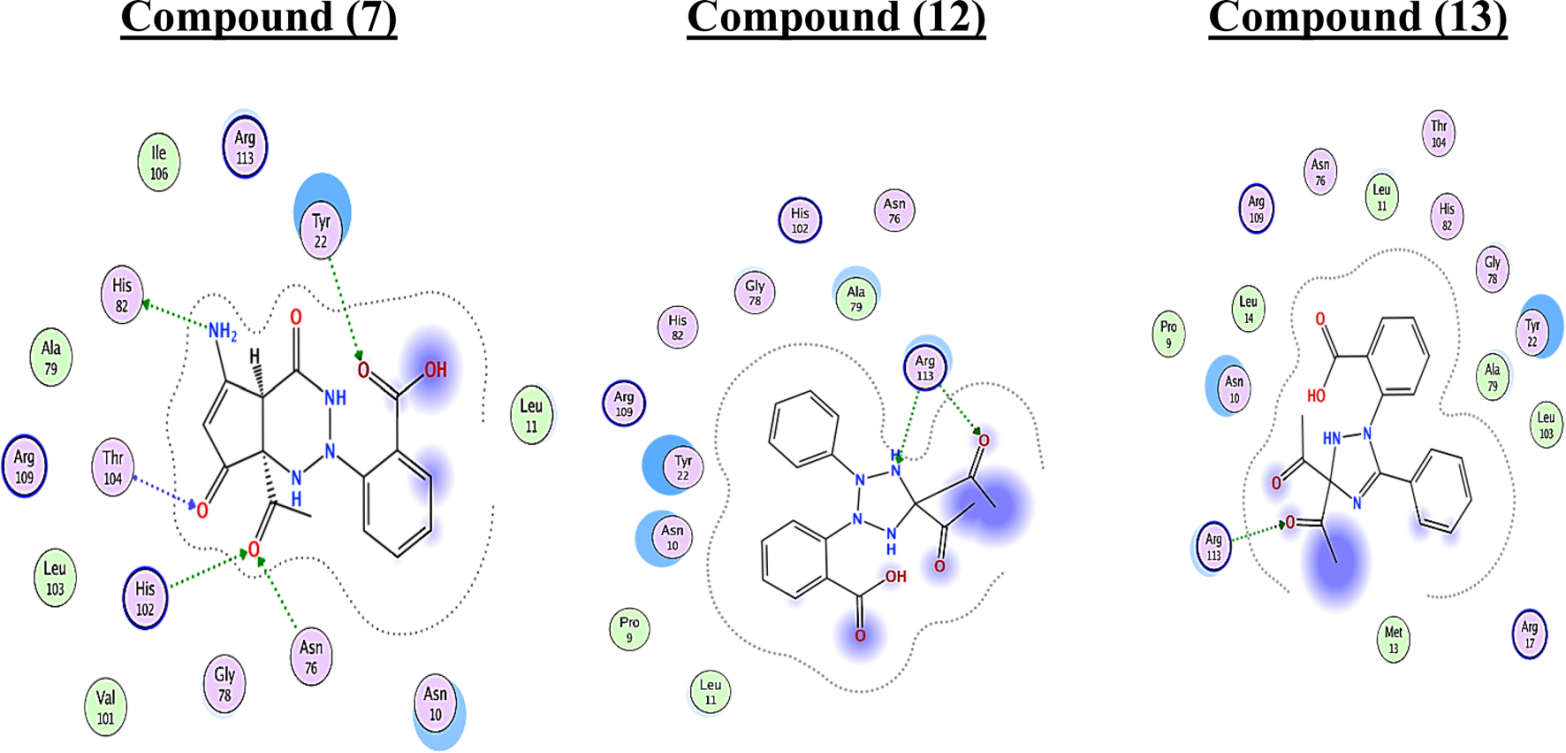
2D receptor interactions of the promising synthetized compounds against *H. Pylori.*

**Figure 17:**
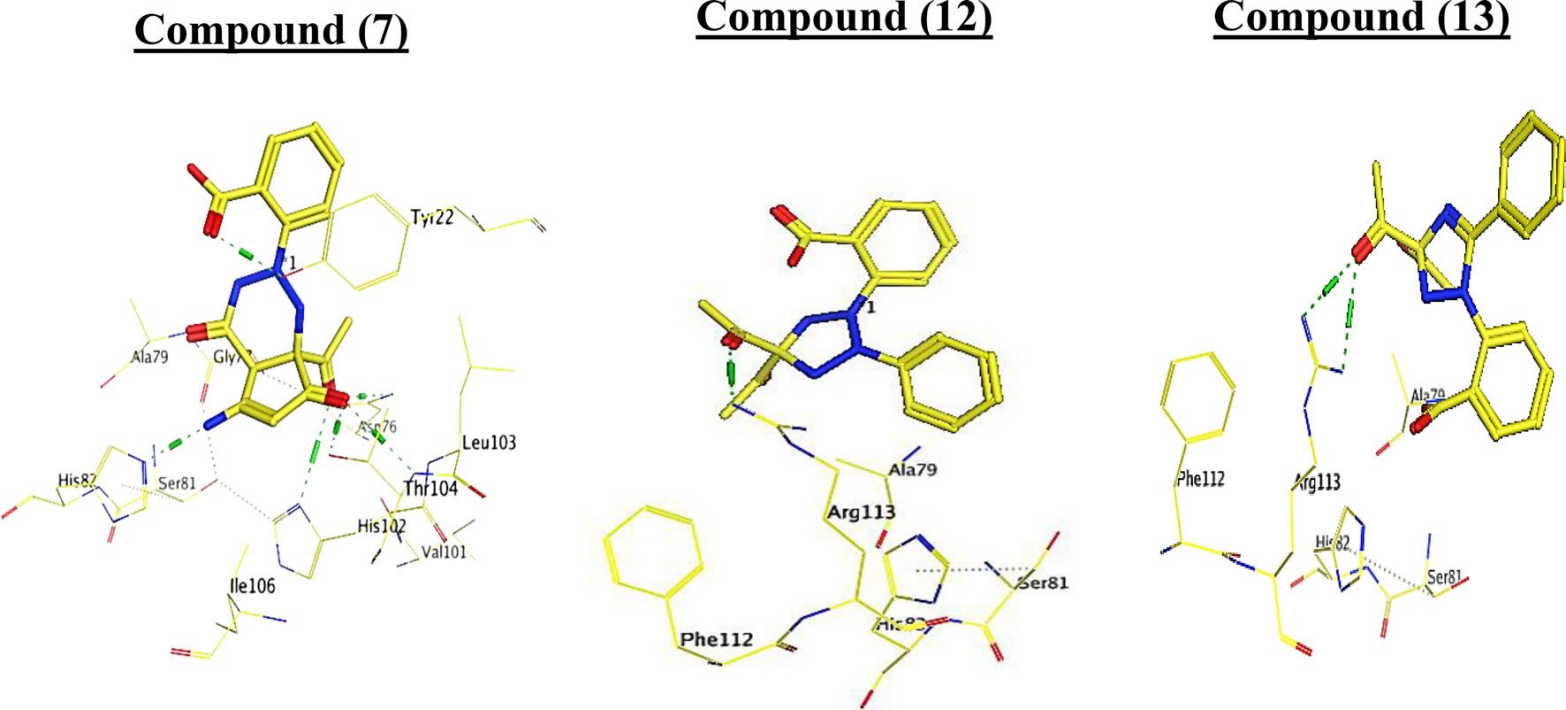
3D receptor interactions of the promising synthetized compounds against *H. Pylori.*

As depicted in Table 7, target 7 was bound to the active site of type II dehydroquinase with docked scores of − 5.75 (Kcal/mol). While 7 formed five H-bonds; three of them between the 2 oxygen of ketone group atom and the side chain of Thr104, His102 and Asn76, and the other was between the NH, OH group and the side chain of His82 and Tyr22, respectively. Whereas compounds 12 and 13 exhibited the highest docking scores [− 6.08 and − 6.34 (Kcal/mol), respectively]. Their re-docking poses were illustrated in Figs. 16, 17. As displayed, compound **12** and **13** formed two H-bond with the Arg113.

**Table 7:** The binding scores, RMSD values, distance, receptor interactions of the most three promising compounds (7, 12 and 13) compared to the reference ligand [JPS] as a reference for bactericidal activity against H. pylori.

Comp	Score (Kcal/mol)	RMSD	Receptor interactions	Distance (Å)	E (Kcal/mol)
Ligand JPS	− 7.3664	2.1414	His82/ H- donor	2.91	− 2.00
			Thr104/ H-acceptor	3.06	− 7.20
			Thr104/ H-acceptor	3.24	− 1.40
			Asn76/ H-acceptor	3.19	− 0.80
			His102/ H-acceptor	2.99	− 4.80
			Arg113/ H-acceptor	3.03	− 0.90
			His102/ Ionic	2.99	− 4.50
7	− 5.7570	1.0563	His82/ H- donor	2.74	− 1.50
			Thr104/ H-acceptor	2.75	− 2.30
			Tyr22/ H-acceptor	2.95	− 2.40
			Asn76/ H-acceptor	2.98	− 1.50
			His102/ H-acceptor	3.40	− 2.70
12	− 6.0893	2.3797	Arg113/ H-acceptor	2.95	− 3.70
			Arg113/ H-acceptor	2.94	31.20
13	− 6.3499	1.9341	Arg113/ H-acceptor	3.01	− 3.00
			Arg113/ H-acceptor	2.95	− 3.10

## Materials and methods

### Preparation of CuO-NPs

As an initiator, a suitable amount of copper nitrate [Cu(NO_3_)_2_] was liquefied in distilled water as a solvent. A reducing agent of 0.1 M sodium hydroxide (NaOH) was gradually added until the pH reached 12 at 50 °C while magnetic stirring (400 rpm) for one hour. The acquired blue precipitate was permitted to stand for 24 h till the color changed from blue to black. The resulting black precipitate was rinsed many times with deionized water until the pH reached 7. As a result, the cleaned precipitate was dehydrated for 6 h in an electric furnace at 100 °C. The produced CuO-NPs were investigated using (XRD), SEM/EDX, and the crystallinity of the nanoparticle was determined using (HR-TEM) and Specific Surface Area (SSA).

### Characterization techniques

CuO-NPs were subjected to XRD, HR-TEM and SEM/EDX to recognize the particle characteristics, which closely coordinated with JCPDC standard. CuO phase identification and phase clarity evaluation were performed using XRD, enhancement of crystalline phases and evaluation of the produced sample's nano-size. XRD was performed using [Bruker D8 advance diffractometer, Germany] with monochromatized Cu K_α_ (λ = 1.542 Å) radiation in the scope of scattering angle (2θ) in the range of 5–80°. The nature and crystallinity of the nanoparticle were verified by high resolution transmission electron microscope [(HR-TEM), Joel model JEM-2100, Japan]. The aqueous dissipation of the particulate was drop-casted onto a copper grid that had been coated with carbon and allowed to air dry at ambient temperature before being carefully considered. The micron-sized structure and appearance of the CuO was presented using scanning electron microscope equipped with energy dispersive x-ray microanalysis (SEM/EDX, model FEJ Quanta 250 Fei, Netherlands) operating at voltage 15 kV. The samples’ surface was coated by gold by a [S150A sputter coater, Edwards, England] under 50 mA current, 0.1 Torr and vacuum 1.2 kV voltage. The gold concealing was to improve the samples’ scanning. In the nanoparticles, the specific surface area (SSA) played an important character, because of the high ratio of surface to volume accompanied by a decrease in the dimension of particle. It has an accurate implication in case of the reactions on surfaces, adsorption, and heterogeneous catalysis. The surface area of the CuO-NPs permitted the overall size of the material significantly influenced the chemical reaction between the CuO and the substrate, which typically takes place at the interface or on the surface. In present research, surface area of CuO-NPs was 58.4419 (m^2^/g), which was restrained via Quantachrome Touch WinTM, model NOVA touch 4LX, using nitrogen as adopant^35^.

### Synthesis

#### Materials and methods

High-quality materials were employed to complete the present research work. Sigma-Aldrich provided all the chemicals (Taufkirchen, Germany) while, El-Nasr Pharmaceutical Chemicals corporation supplied all solvents (analytical reagent grade, Egypt). The melting points were tested using a Cole-Parmer numerical Electrothermal IA 9100 Series equipment (Beacon Road, Stone, Staffordshire, ST15 OSA, UK) are unrequited. C, N, and H considerations were implemented using a PerkinElmer CHN 2400. IR spectra were documented on Pye-Uniearn using KBr wafer technique and Beckman spectrophotometers in Mansoura university. For generating ^1^H spectra, a Bruker 400, 100 MHz NMR Spectrometer was utilized, DMSO-d_6_ was used as a solvent, and chemical shifts were represented in (ppm) in the Main Laboratories of Chemical war, Nasr city, Egypt. The ^13^C-NMR spectra were recorded on ECA 500 MHz in Mansoura university. Thin-layer chromatography (TLC) sheets coated with UV fluorescent silica gel Merck 60 F254 plates were used to monitor the reactions, which were observed using a UV laser and various solvents as mobile phases. CuO-NPs was provided by National Research Centre (NRC). High Resolution-Transmission Electron Microscope (HR-TEM) tested via JEM-2100 (JEOL, Tokyo, Japan) Electron microscopy unit- National Research Centre. The morphology and microcrystalline structure were presented by a scanning electron microscope stimulated with energy dispersive x-ray microanalysis (SEM/EDX, model FEJ Quanta 250 Fei, Netherlands). The X-ray diffraction (XRD) configuration was presented by means of D8 Advance diffractometer (Bruker, Germany) by National Research Centre. Specific surface area (SSA) was sustained by Quantachrome Touch Win™, model NOVA touch 4LX. *Anti-H. Pylori* activity was performedat Pharmacology, Faculty of Veterinary Medicine, Cairo University, Egypt. Molecular docking studies were applied by using MOE software (2015).

#### Synthesis of Compound 2 using CH_3_COONa

Anthranilic acid solution [an aromatic acid with the formula C_6_H_4_(NH_2_)(CO_2_H)](0.274 g, 0.002 mol) within (0.2 mL conc. HCl and 3 mL ice distilled water), the solution of NaNO_2_ (0.138 g, 0.002 mL) in (1 mL ice distilled water) added drop by drop then acetylacetone (0.200 g, 0.192 mL, 0.001 mol) was used in the mixture of sodium acetate (0.1 g) in (1 mL ice distilled water). The reaction mixture was well mixed on ice path, for one hour, then examined by TLC system via methylene chloride as eluent, a yellow residue solid of compound **2**: 97%, m.p.: ›300 °C was established, collected and recrystallized from ethanol.

#### Fabrication of Compound 2 using CuO-NPs

To a solution of diazonium salt, supplement (10 mmol) of CuO-NPs then acetylacetone (C_5_H_8_O_2_) was added (0.200 g, 0.192 mL, 0.001 mol) gradually. Using methylene chloride (CH_2_Cl_2_) as the fluid, the reaction process was monitored by chromatography while the mixture was agitated on an ice bath for four minutes. The generated solid was then collected and crystallized from ethanol as a yellow powder of compound **2**: 99%, m.p.: ›300 °C.

##### 2'-((2,4-dioxopentane-3,3-diyl) bis(diazene-2,1-diyl)) dibenzoic acid (2)

IR (KBr, ν, cm^−1^): 3444–3360 (broad band for 2 OH), 3051–2839 (CH for aromatic and aliphatic), 1678 (C=O for 2 carboxylic groups), 1624 (C=O for 2 ketones), 1450 (2 N=N). ^1^H-NMR (DMSO-d_6_, 400 MHz): *δ* = 2.421 (s,6*H*, 2CH_3_ of C=O), 7.121- 7.161 (t, 2*H*, *J* = *8*, *H*_Aryl_), 7.491- 7.533 (t, 2*H*, *J* = *8.4*, H_Aryl_), 7.867- 7.888 (d, 2*H*, *J* = *8.4*, *H*_Aryl_), 7.955–7.974 (d, 2*H*, *J* = *7.6*, *H*_Aryl_), 15.842 (s,2*H*, 2OH exchangeable by D_2_O).^13^C-NMR (DMSO-d_6_, 100 MHz): *δ* = 26.84(2CH_3_), 115.04(2Phenyl carbon), 124.07(C), 125.79, 131.04, 131.72, 134.10, 143.82 (2Phenyl carbon), 168.81(2COOH), 194.15, 197.29 (2C=O). MS (*M/Z*): M^+*•*^(396.56), Base-peak (207.28). Anal.Calcd. for C_19_H_16_N_4_O_6_ (396.36): C,57.58; H, 4.07; N, 14.14; Found; C,57.56; H, 4.06; N, 14.12%.

##### 2-(4,4-diacetyl-7,7-dimethyl-5-oxo-3,4,5,6,7,8-hexahydro-2*H*-benzo[*e*][1,2,3]oxadiazin-2-yl)benzoic acid (3)

In the presence of a few drops of trimethylamine (TEA), compound **2** solutions (2.00 g, 0.005 mol) in (25 mL) dimethylformamide (DMF, C_3_H_7_NO) were monitored by the addition of dimadone (0.70 g, 0.005 mol). The mixture was allowed to reflux for 17 h before being monitored using a thin-layer chromatography (TLC) apparatus with methylene chloride as an eluent. After allowing it to cool overnight, it was left to reflux for 17 h before being gradually poured over ice cubes and filtered. The separated solid was recrystallized using a mixture of DMF and ethanol by the ratio (2:1) to yield brown powder of compound **3**: 87%, m.p.: 222 °C. IR (ν, cm^−1^): 3451 (OH), 3082(NH), 3004(CH for aromatic), 2928(CH for aliphatic), 1717 (C=O of ketone), 1701 (C=O of acid). ^1^H-NMR (DMSO-d_6_, 400 MHz): *δ* = 2.434 (s, 6*H*, 2CH_3_), 2.451 (s, 6*H*, 2CH_3_), 2.705 (s, 2*H*, CH_2_), 2.865 (s, 2*H*, CH_2_), 7.218- 7.256 (t, 1*H*, *J* = 7.6, *H*_Aryl_), 7.660–7.706 (t, 1*H*, *J* = *8.4.*, *H*_Aryl_), 7.910–7.920(d, 1*H*, *J* = *8*, *H*_Aryl_), 7.946(s,1*H*, NH),7.964–7.984 (d, 1*H*, *J* = *8*, *H*_Aryl_), 15.145(s,1*H*, OH).^13^C-NMR (DMSO-d_6_, 100 MHz): *δ* = 26.63 (2CH_3_), 31.24(2CH_3_), 36.63(CH_2_), 43.20(CH_2_), 50.70 (C), 92.07(C), 115.52, 116.30(C=C), 124.068, 131.45, 134.58, 134.70, 143.57, 167.93(Phenyl carbon), 195.680(C=O), 196.85(C=O), 200.30(C=O). Anal.Calcd. for C_20_H_22_N_5_O_6_ (386.40): C,62.17; H, 5.74; N, 7.25; Found; C,62.68; H, 5.71; N, 7.56%.

##### 2-(4,4-diacetyl-5-methyl-7-phenyl-4,7-dihydropyrazolo[4,3-*e*][1,2,3]oxadiazin-2(3*H*)-yl)benzoic acid (4)

A few droplets of triethylamine (1 mL), a combination of compound **2** (0.5 g, 0.0012 mol) and phenylhydrazone (0.2088g, 0.0012 mol) was dissolved in (18 mL) dimethylformamide. After 13 h of thermal reaction, this reaction was monitored by TLC system using methylene chloride as eluent, the solution was chilled and discarded over smashed ice. A solution of mixed DMF/ethanol by ratio (2:1) was used to assemble the residue and recrystallize it to emanate an orange powder of compound **4**: 59%, m.p.: 216–200℃. IR (ν, cm^-1^): 3450 (OH), 3080(NH), 2974(CH for aliphatic), 1718 (C=O of ketone), 1700 (C=O of acid). ^1^H-NMR (DMSO-d_6_, 400 MHz): *δ* = 2.441 (s, 6*H*, 2CH_3_), 2.459 (s, 3*H*, CH_3_), 7.222- 7.253 (t, 1*H*, *J* = 7.2, *H*_Aryl_), 7.260–7.270(d, 1*H*, *J* = *8*, *H*_Aryl_), 7.675–7.710 (t, 1*H*, *J* = *8.4.*, *H*_Aryl_), 7.723–7.737 (d, 1*H*, *J* = *8*, *H*_Aryl_), 7.931(s,1*H*, NH),7.966–7.016(m, 5*H*, *H*_Aryl_), 15.148 (s,1*H*, OH).^13^C-NMR (DMSO-d_6_, 100 MHz): *δ* = 26.63 (2CH_3_), 31.24(CH_3_), 100.30, 104.24, 108.52, 108.30, 115.52, 116.25, 124.07, 131.44, 134.60, 134.69, 143.56, 148.24, 152.63, 167.82(C=O), 196.85(C=O). Anal.Calcd. for C_22_H_20_N_4_O_5_ (420.43): C,62.85; H, 4.40; N, 13.33; Found; C,62.92; H, 4.36; N, 13.30%.

##### 2-(4-acetyl-4-(methoxy-l2-methyl)-5,7-dioxo-3,4,5,6,7,8-hexahydro-2*H*-pyrimido[5,4-*e*][1,2,3]oxadiazin-2-yl)benzoic acid (5)

A mixture of compound **2** (0.5g, 0.0012 mol), barbituric acid (C_4_H_4_N_2_O_3_) (0.153 g, 0.00012 mol) and few drops of triethylamine in (13 mL) dimethylformamide was refluxed for 17 h and the reaction was monitored by TLC system using methylene chloride as eluent. The mixture was cooled and poured over ice cold water. The set up solid was strained, dried at room temperature before being recrystallized using a mixture of DMF/ethanol by ratio (2:1) to give out compound **5** as yellow powder: 68%, m.p.:230℃. IR (ν, cm^-1^): 3448 (OH), 3081(NH), 3013(CH for aromatic), 2930(CH for aliphatic),1717 (C = O of ketone), 1702 (C=O of acid), 1678 (C=O of amide). ^1^H-NMR (DMSO-d_6_, 400 MHz): *δ* = 2.431 (s, 3*H*, CH_3_), 2.446 (s, 3*H*, CH_3_), 7.211–7.249 (t, 1*H*, *J* = 7.7, *H*_Aryl_), 7.645–7.694 (t, 1*H*, *J* = *8.*, *H*_Aryl_), 7.960(s,1*H*, NH),7.969–7.978(d, 1*H*, *J* = *8*, *H*_Aryl_), 7.999–8.020 (d, 1*H*, *J* = *8.4*, *H*_Aryl_), 10.241(s,1*H*, NH),10.512(s,1*H*, NH), 15.190(s,1*H*, OH).^13^C-NMR (DMSO-d_6_, 100 MHz): *δ* = 26.63 (2CH_3_), 74.50, 92.07, 115.52, 116.23, 124.07, 131.44, 134.60, 134.69, 143.56, 167.91 (C=O),172.00(C=O), 195.68(C=O), 196.84(C=O). Anal.Calcd. for C_16_H_14_N_4_O_7_ (374.31): C,51.34; H, 3.77; N, 14.97; Found; C,61.30; H, 3.75; N, 14.95%.

##### (E)-2-(7a-acetyl-5-amino-3-(benzylideneamino)-4,7-dioxo-1,3,4,4a,7,7a-hexahydro-2*H*-cyclopenta[*d*][1,2,3]triazin-2-yl)benzoic acid (6)

Compound **2** (0.5 g, 0.0012 mol), hydrazone (0.223 g, 0.0012 mol), and a few drops of triethylamine were heated under reflux condition for 13 h in dimethylformamide (20 mL) and the reaction was monitored by TLC system using methylene chloride as eluent. The mixture was allowed to refresh before being dumped into ice water. The resulting solid was filtered and dried at room temperature before being recrystallized with mixture of DMF and ethanol by ratio (2:1) to yield compound **6** as black powder: m.p.:130–134 ℃. IR (ν, cm^−1^): 3426 (OH), 3216(NH), 2927(CH for aliphatic), 1717 (C=O of ketone), 1702 (C=O of acid), 1674 (C=O of amide), 1600 (N=N). ^1^H-NMR (DMSO-d_6_, 400 MHz): *δ* = 2.429 (s, 3*H*, CH_3_), 3.979(s, 1H, CH), 5.387(s, 1H, CH), 7.183- 7.221 (t, 1*H*, *J* = 7.6, *H*_Aryl_), 7.340(s,1*H*, NH), 7.378(s,1*H*, NH_2_), 7.480–7.484 (t, 1*H*, *J* = *8.*, *H*_Aryl_), 7.716 (m, 5*H*, *H*_Aryl_), 7.722 (s,1*H*, CH), 7.925–7.944(d, 1*H*, *J* = *7.6*, *H*_Aryl_), 7.964–7.978 (d, 1*H*, *J* = *8*, *H*_Aryl_), 15.385(s,1*H*, OH).^13^C-NMR (DMSO-d_6_, 100 MHz): *δ* = 26.62, 35.83, 116.35, 116.91, 124.04, 131.19, 131.45, 133.76, 134.63, 143.45, 151.55, 162.37,168.07, 169.65, 195.59, 196.85. Anal.Calcd. for C_22_H_19_N_5_O_5_ (433.42): C,60.97; H, 4.42; N, 16.16; Found; C,60.95; H, 4.39; N, 16.13%.

##### 2-(7a-acetyl-5-amino-4,7-dioxo-1,3,4,4a,7,7a-hexahydro-2*H*-cyclopenta[*d*][1,2,3]triazin-2-yl)benzoic acid (7)

A few drops of triethylamine, was added to the solution of compound **2** (2 g, 0.005 mol) in (18 mL) dimethylformamide was added to cyanoacetamide (0.5 g, 0.005 mol). The mixture was refluxed for 13 h, the reaction was monitored by TLC system using methylene chloride as eluent, then overnight cooling, followed by gradual pouring through crushed snow and filtering. The precipitate was dried and recrystallized with mixture of DMF and ethanol by ratio (2:1) to produce compound **7** as brown powder: 57%, m.p.:213℃. IR (ν, cm^−1^): 3453 (OH), 3018(broad band of NH_2_ and NH), 1722 (C=O of ketone), 1702 (C=O of acid), 1638 (C=O of amide). ^1^H-NMR (DMSO-d_6_, 400 MHz): *δ* = 2.439 (s, 3*H*, CH_3_), 4.331(s, 1H, CH), 5.341(s, 1H, CH), 7.218- 7.255 (t, 1*H*, *J* = 8, *H*_Aryl_), 7.671–7.707 (t, 1*H*, *J* = *7.2.*, *H*_Aryl_), 7.930(s,1*H*, NH), 7.965–7.968(d, 1*H*, *J* = *8*, *H*_Aryl_), 7.989–8.012 (d, 1*H*, *J* = *8.4*, *H*_Aryl_), 8.149(s,1*H*, NH_2_), 8.167 (s,1*H*, NH), 15.150(s,1*H*, OH).^13^C-NMR (DMSO-d_6_, 100 MHz): *δ* = 26.62, 31.24, 115.48, 116.41, 124.06, 131.44, 134.55, 134.66, 143.55, 167.94, 175.21, 180.11,195.63, 196.83. Anal. Calcd. for C_15_H_14_N_4_O_5_ (330.30): C,54.55; H, 4.27; N, 16.96; Found; C,54.51; H, 4.24; N, 16.93%.

##### 2-(7a-acetyl-3,5-diamino-4,7-dioxo-1,3,4,4a,7,7a-hexahydro-2*H*-cyclopenta[*d*][1,2,3]triazin-2-yl)benzoic acid (8)

Compound **2** (2 g, 0.005 mol) was dissolving in (9 mL) dimethylformamide then was mixed with a solution of cyanoaceto hydrazide (convenient intermediate for the production of diversity of heterocyclic compounds) (0.49 g, 0.005 mol) in (9 mL) dimethyl formamide in the occurrence of few drops of triethylamine (1 ml). The reaction components were heated under reflux for 17 h, the reaction was followed by TLC system using mixture of methylene chloride as eluent. The dissociated crystal was filtered out and crystallized by a combination of DMF and ethanol by ratio (2:1) after being refrigerated for a night and poured progressively over broken ice by ratio (2:1) to give off compounds **8** as black powder: 78%, m.p.:237 °C. IR (ν, cm^−1^): 3449 (OH), 3070(broad band of NH_2_ and NH), 1717 (C=O of ketone), 1702 (C = O of acid), 1676 (C=O of amide). ^1^H-NMR (DMSO-d_6_, 400 MHz): *δ* = 2.422 (s, 3*H*, CH_3_), 4.314(s, 1H, CH), 5.067(s, 1H, CH), 6.452- 6.489 (t, 1*H*, *J* = 6.8, *H*_Aryl_), 6.694–6.714(d, 1*H*, *J* = *8*, *H*_Aryl_), 7.558–7.567 (d, 1*H*, *J* = *8*, *H*_Aryl_), 7.644–7.682 (t, 1*H*, *J* = *7.6.*, *H*_Aryl_), 7.926(s,1*H*, NH_2_), 7.954(s,1*H*, NH), 7.973 (s,1*H*, NH_2_),15.150(s,1*H*, OH).^13^C-NMR (DMSO-d_6_, 100 MHz): *δ* = 26.62, 35.83, 116.35, 116.91, 124.05, 131.19, 131.46, 133.77, 134.37, 143.54,151.56, 162.37, 168.08,195.59, 196.85. Anal.Calcd. for C_15_H_15_N_5_O_5_ (345.32): C,52.17; H, 4.38; N, 20.28; Found; C,52.13; H, 4.35; N, 20.26%.

##### 2-(7a-acetyl-5-methyl-4,7-dioxo-3-phenyl-1,3,4,4a,7,7a-hexahydro-2*H*-cyclopenta[*d*][1,2,3]triazin-2-yl)benzoic acid (9)

Compound **2** (0.50 g, 0.0012 mol) and 3-oxo-*N*- phenyl butanamide (0.212 g, 0.0012 mol) were dissolved in (20 mL) dimethylformamide in presence of few drops of triethylamine (1 mL), The TLC system used a mixture of methylene chloride as the eluent for observing the reaction. After cooling and being poured over ice that had been crushed, the fluid was refluxed for 15 h. To create compounds **9**, the produced precipitate was collected and crystallized with a solution of DMF and ethanol in a 2:1 ratio, yellow powder of compound **9**: 76%, m.p.:224 °C. IR (ν, cm^−1^): 3449 (OH), 3096(broad band of NH), 3003(CH for aromatic), 2931(CH for aliphatic), 1727 (C=O of ketone), 1703 (C=O of acid), 1680 (C=O of amide). ^1^H-NMR (DMSO-d_6_, 400 MHz): *δ* = 2.439 (s, 3*H*, CH_3_), 2.458 (s, 3*H*, CH_3_), 4.331(s, 1H, CH), 6.340(s, 1H, CH), 7.220- 7.258 (t, 1*H*, *J* = 7.2, *H*_Aryl_), 7.671—7.710 (t, 1*H*, *J* = *7.2.*, *H*_Aryl_), 7.930(s,1*H*, NH), 7.965–8.034 (m, 5*H*, *H*_Aryl_), 8.140–8.146 (d, 1*H*, *J* = *8*, *H*_Aryl_), 8.167–8.177(d, 1*H*, *J* = *8*, *H*_Aryl_), 15.150(s,1*H*, OH).^13^C-NMR (DMSO-d_6_, 100 MHz): *δ* = 26.62, 31.24, 115.48, 116.42, 124.07, 131.45, 134.52, 134.66, 143.55, 151.56, 162.37,167.94, 168.08, 169.65, 195.63, 196.84. Anal.Calcd. for C_22_H_19_N_3_O_5_ (405.41): C,65.18; H, 4.72; N, 10.37; Found; C,65.15; H, 4.70; N, 10.33%.

##### 2-(7a-acetyl-4,5-dimethyl-7-oxo-7,7a-dihydrocyclopenta[*d*][1,2,3]oxadiazin-2(1*H*)-yl)benzoic acid (10)

In the existence of a few drops of triethylamine, a solution of compound **2** (2.00 g, 0.005 mol) in (22 mL) dimethylformamide was add the following acetyl acetone (0.50ml, 0.005 mol). The reaction mixture was heated for 17 h, the reaction was monitored by TLC system using methylene chloride as eluent, then cooled overnight before being dropped steadily over ice cubes and filtered. The separated solid was recrystallized by a mixture of DMF and ethanol by the ratio (2:1) to produce brown powder of compound **10**: 85%, m.p.: 184℃. IR (ν, cm^−1^): 3451 (OH), 3085(NH), 3005(CH for aromatic), 2929(CH for aliphatic), 1723 (C=O of ketone), 1705 (C=O of acid). ^1^H-NMR (DMSO-d_6_, 400 MHz): *δ* = 1.324 (s, 3*H*, CH_3_), 2.429 (s, 3*H*, CH_3_), 2.438 (s, 3*H*, CH_3_), 6.340 (s, *H*, CH), 7.166- 7.204 (t, 1*H*, *J* = 7.2, *H*_Aryl_), 7.577–7.616 (t, 1*H*, *J* = *7.6.*, *H*_Aryl_), 7.925(s,1*H*, NH),7.947–7.951(d, 1*H*, *J* = *8*, *H*_Aryl_), 7.967–7.971 (d, 1*H*, *J* = *8*, *H*_Aryl_), 15.516(s,1*H*, OH).^13^C-NMR (DMSO-d_6_, 100 MHz): *δ* = 26.59, 31.22, 115.15, 123.91, 131.49, 133.51, 134.46, 143.49, 151.56, 162.37, 168.15, 195.26, 196.84. Anal. Calcd. for C_17_H_16_N_2_O_5_ (328.32): C,62.19; H, 4.91; N, 8.53; Found; C,62.15; H, 4.87; N, 8.50%.

##### 2-(7a-acetyl-5-hydroxy-4-methyl-7-oxo-7,7a-dihydrocyclopenta[*d*][1,2,3]oxadiazin-2(1*H*)-yl)benzoic acid (11)

Compound **2** (2.00 g, 0.005 mol), ethyl acetoacetate (0.63 mL, 0.005 mol), and a few drops of trimethylamine [(N(CH_2_CH_3_)_3_] were heated under reflux condition for 21 h in dimethylformamide (22 mL) and the reaction was monitored by TLC system using methylene chloride as eluent. The mixture was allowed to cool before being dumped into ice water. The resultant solid was filtered and dried at room temperature before being recrystallized with mixture of DMF and ethanol by ratio (2:1) to yield compound **11** as yellow powder: 76%, m.p.:248℃. IR (ν, cm^−1^): 3449 (OH), 3082(NH), 3005(CH for aromatic), 2927(CH for aliphatic), 1727 (C=O of ketone), 1708 (C=O of acid). ^1^H-NMR (DMSO-d_6_, 400 MHz): *δ* = 2.429 (s, 3*H*, CH_3_), 2.438 (s, 3*H*, CH_3_), 6.340(s, 1H, CH), 7.166- 7.204 (t, 1*H*, *J* = 7.2, *H*_Aryl_), 7.577–7.616 (t, 1*H*, *J* = *7.6.*, *H*_Aryl_), 7.925(s,1*H*, NH), 7.947–7.951(d, 1*H*, *J* = *8*, *H*_Aryl_), 7.967–7.971 (d, 1*H*, *J* = *8*, *H*_Aryl_), 11.570(s,1*H*, OH), 15.516(s,1*H*, OH).^13^C-NMR (DMSO-d_6_, 100 MHz): *δ* = 26.62, 31.24, 100.30, 104.24, 115.52, 116.26, 124.08, 131.45, 134.60, 134.70, 143.57, 151.55, 162.37, 167.92, 195.69, 196.85, 200.30. Anal.Calcd. for C_16_H_14_N_2_O_6_ (330.30): C,58.18; H, 4.27; N, 8.48; Found; C,58.14; H, 4.25; N, 8.44%.

##### 2-(5,5-diacetyl-3-phenyltetrazolidin-2-yl)benzoic acid (12)

Compound **2** (0.02 mol), phenyl hydrazine (0.02 mol), and a few drops of triethylamine were heated in 21 mL of dimethylformamideunder reflux conditions for 17 h, and the process was monitored using a TLC apparatus with methylene chloride as the eluent. Before being poured into freezing water, the mixture was cooled completely. The resultant product was separated and dried at room temperature before even being recrystallized using a 2:1 combination of water and ethanol to obtain compound **12** as a black crystalline: 56%, m.p.:130℃. IR (ν, cm^-1^): 3438 (OH), 3057(NH), 2922(CH for aliphatic), 1724 (C=O of ketone), 1710 (C=O of acid). ^1^H-NMR (DMSO-d_6_, 400 MHz): *δ* = 2.426 (s, 6*H*, 2CH_3_), 6.450- 6.487 (t, 1*H*, *J* = 7.2, *H*_Aryl_), 6.692–6.713(d, 1*H*, *J* = *8.4*, *H*_Aryl_), 7.913–7.974 (m, 5*H*, *H*_Aryl_), 8.034–8.164 (t, 1*H*, *J* = *8.*, *H*_Aryl_), 8.201–8.219 (d, 1*H*, *J* = *7.2*, *H*_Aryl_), 10.214(s,1*H*, NH), 10.837(s,1*H*, NH), 15.351(s,1*H*, OH).^13^C-NMR (DMSO-d_6_, 100 MHz): *δ* = 26.59, 128.35, 129.21, 129.34, 129.42, 129.51, 131.49, 133.75, 141.52, 143.87, 151.56, 179.88, 196.83. Anal.Calcd. for C_18_H_18_N_4_O_4_ (354.37): C,61.01; H, 5.12; N, 15.81; Found; C,59.98; H, 5.10; N, 15.78%.

##### 2-(3,3-diacetyl-5-phenyl-2,3-dihydro-1*H*-1,2,4-triazol-1-yl)benzoic acid (13)

A combination of compound **2** (2 g, 0.005 mol) in (18 mL) dimethyl formamide was mixed to benzyl amine in the existence of a few drops of triethylamine (0.54 ml, 0.005 mol). The mixture was boiled for 13 h, then cool overnight before even being poured step by step over ice cubes and monitored using a TLC apparatus with methylene chloride as the eluent. The precipitate was dried and recrystallized using a 2:1 combination of DMF and ethanol to yield compound **13** as a brown powder 76% with a melting point of 228°C. IR (ν, cm^−1^): 3451 (OH), 3078(NH), 3003(CH for aromatic), 2928(CH for aliphatic),1717 (C=O of ketone), 1708 (C=O of acid), 1600 (C=N). ^1^H-NMR (DMSO-d_6_, 400 MHz): *δ* = 2.442 (s, 6*H*, 2CH_3_), 7.220- 7.258 (t, 1*H*, *J* = 7.6, *H*_Aryl_), 7.671—7.709 (t, 1*H*, *J* = *8.*, *H*_Aryl_), 7.760–7.882 (m, 5*H*, *H*_Aryl_), 7.930(s,1*H*, NH), 7.967–7.989(d, 1*H*, *J* = *8.8*, *H*_Aryl_), 8.013–8.041 (d, 1*H*, *J* = *8*, *H*_Aryl_), 15.147(s,1*H*, OH).^13^C-NMR (DMSO-d_6_, 100 MHz): *δ* = 26.59, 98.61, 115.35, 117.30, 124.06, 131.46, 134.12, 134.53, 143.51, 158.54, 162.35, 168.11, 195.46, 196.82. Anal.Calcd. for C_19_H_17_N_3_O_4_ (351.36): C, 64.95; H, 4.88; N, 11.96; Found; C, 64.91; H, 4.85; N, 11.95%.

##### 2-((1-acetyl-2,2-dicyano-5-oxo-3-phenylcyclopent-3-en-1-yl)diazenyl)benzoic acid (14):

In the existence of a few drops of triethylamine, a solution of compound **2** (0.50 g, 0.0012 mol) in (11 mL) dimethylformamide was mixed with a solution of benzilidine malononitrile (0.185 g, 0.0012 mol) in (11 mL) dimethylformamide. The resulting mixture was refluxed for 17 h, the interaction was monitored by TLC process using methylene chloride as eluent, then allowing it to cool for a night and poured gradual over ice cubes, the isolated crystal was filtered off and recrystallized by mixture of DMF and ethanol by ratio (2:1) to give off compound **14** as black powder: 88%, m.p.:148 °C. IR (ν, cm^−1^): 3449 (OH), 3060(CH for aromatic), 2932(CH for aliphatic), 2199 (CN), 1727 (C=O of ketone), 1708 (C=O of acid), 1493 (N=N). ^1^H-NMR (DMSO-d_6_, 400 MHz): *δ* = 2.425 (s, 3*H*, CH_3_), 5.364 (s, 1H, CH), 7.208–7.246 (t, 1*H*, *J* = 7.2, *H*_Aryl_), 7.655–7.664 (t, 1*H*, *J* = *7.6.*, *H*_Aryl_), 7.920- 7.991 (m, 5*H*, *H*_Aryl_), 8.098–8.110(d, 1*H*, *J* = *8*, *H*_Aryl_), 8.128–8.146 (d, 1*H*, *J* = *7.2*, *H*_Aryl_), 15.126(s,1*H*, OH).^13^C-NMR (DMSO-d_6_, 100 MHz): *δ* = 30.80, 31.25, 98.61, 115.47, 116.26, 124.05, 128.26, 128.45, 128.82, 131.43, 134.56, 143.54, 158.54, 162.35, 167.91, 195.64, 196.83. Anal. Calcd. for C_22_H_14_N_4_O_4_ (398.38): C,66.33; H, 3.54; N, 14.06; Found; C,66.29; H, 3.50; N, 11.03%.

##### 2-((2-acetyl-3-oxo-1,5-diphenyl-2,3-dihydro-1*H*-pyrrol-2-yl)diazenyl)benzoic acid (15)

In (15 mL) dimethyl formamide, a mixture of compound **2** (0.5 g, 0.0012 mol), benzal aniline (0.227 g, 0.00012 mol), and a few drops of triethylamine was refluxed for 13 h and the reaction was monitored using a TLC apparatus with methylene chloride as the eluent. After cooling, the mixture was poured over ice cold water. The created solid was filtered and dried at room temperature before being recrystallized by a 2:1 mixture of DMF and ethanol to yield compound 1**5** as a black powder with a melting point of 100–112 °C. IR (ν, cm^-1^): 3366 (OH), 3028(CH for aromatic), 2926(CH for aliphatic), 1730 (C=O of ketone), 1704 (C = O of acid), 1492 (N=N). ^1^H-NMR (DMSO-d_6_, 400 MHz): *δ* = 2.440 (s, 3*H*, CH_3_), 4.433 (s, 1H, CH), 7.174–7.254 (m, 5*H*, *H*_Aryl_), 7.288–7.357 (m, 5*H*, *H*_Aryl_), 7.667–7.686(d, 1*H*, *J* = *7.6*, *H*_Aryl_), 7.706–7.768 (d, 1*H*, *J* = *8*, *H*_Aryl_), 7.790- 7.988 (t, 1*H*, *J* = 8, *H*_Aryl_), 8.010–8.091 (t, 1*H*, *J* = *8*, *H*_Aryl_), 15.166(s,1*H*, OH). Anal.Calcd. for C_25_H_19_N_3_O_4_ (425.44): C, 70.58; H, 4.50; N, 9.88; Found; C, 70.55; H, 4.45; N, 9.84%.

### *Anti-H. pylori* activity

#### Bacterial suspensions production

In susceptibility tests, each strain inoculum was arranged by relocating fresh colonies of the microorganisms into tubes comprising antiseptic physiological saline solution and modifying the turbidity to the 2.0 McFarland standards^36^. This turbidity yields a suspension that relates to approximately 1.0 × 10^8^ CFU/mL of *H. pylori*.

Verification of *anti-H. Pylori* action: The in vitro *anti-H. pylori* activities were established through well agar diffusion method^37^. Temporarily, 100 μL of *H. pylori* suspension (1.0 × 10^8^ colony forming units (CFUs)/mL) was laid out onto Mueller Hinton agar plates (BBL) holding 10% sheep blood. At that time, a ditch of 6–8 mm diameter is perforated using a sterile cork borer, and a 100 μL volume of the antimicrobial agent otherwise solution extract at chosen concentration is presented into the well. The negative control is dimethyl sulfoxide (DMSO), whereas the positive ones are clarithromycin (CLR, 0.05 mg/mL), antibiotics amoxicillin (AMX, 0.05 mg/ml) and metronidazole (MTZ, 0.8 mg/mL). Afterward, maturation of 72 h at 37 °C under a microaerophilic condition by means of humidity, the inhibition zone diameter (IZD) was set on.

##### Minimal inhibitory concentration (MIC)

The micro-dilution broth method, using Mueller–Hinton broth supplemented with lysed horse blood, allowing for the determination of the minimal inhibitory concentration (MIC) of the tested samples. Serial two-fold dilutions were made in order to obtain final concentrations of the tested samples, which ranged from 0.98 to 1000 μg/mL. The sterile 96-well polystyrene microtitrate plates were prepared by dispensing 200 μL of appropriate dilution of the tested samples in broth medium per well. The inocula were prepared with fresh microbial cultures in sterile 0.85% NaCl to match the turbidity of 1.0 McFarland standard, and 2 µL were added to the wells to obtain a final density of 3.0 × 10^6^ CFU (colony forming units)/mL. After incubation at 35 °C for 72 h under microaerophilic conditions (15% CO_2_), the MICs were assessed visually as the lowest concentration of the tested samples showing complete growth inhibition of the reference strain. A positive control (containing inoculum without the tested samples) and a negative control (containing the tested samples without inoculum) were included on each microplate (“Supplementary Information”).

##### Minimal bactericidal concentration (MBC)

MBC was determined by sub-culturing 100 mL of the microbial culture from each well that showed thorough growth inhibition, from the last positive and from the growth control, onto the plates of Mueller–Hinton agar supplemented with 5% horse blood. The plates were incubated at 35 °C for 72 h under microaerophilic conditions, and the MBC was defined as the Lowes concentration of the tested samples without growth of microorganisms. To govern the bactericidal or bacteriostatic effect of the evaluated samples, the ratio of MBC/MIC was considered^38–40^.

##### Statistical analysis

Statistical analysis was performed using SPSS-26 statistical software (SPSS Inc., Chicago, IL, USA). Inhibition zones (mm) were obtained as mean ± *SEM* (One Way Analysis of Variance, ANOVA, Tukey's post-hoc test). The significance level was set at a probability value of less than 0.05 (*p* ˂ 0.05)^39–42^.

### Insilco studies

The tools used for POM analysis, especially OSIRIS, MOLINSPIRATION^39^, ProTox-II^40^ and Pred-hERG^41–44^, operate using the same basic concept. The molecular structure is initially determined either by sketching it or by entering its SMILES (simplified molecular input line entry system) code. The specific parameters are then determined using the fragment system^45–48^, then the software of the molecular operating environment was used to perform the molecular modeling for the highest active compounds^49–78^.

## Conclusion

Aneco-friendly and cost-effective, engagement has been evolved for the fabrication of 2,2′-((2,4-dioxopentane-3,3-diyl) bis(diazene-2,1-diyl) dibenzoic acid **2** in the presence of CuO-NPs to start a fast and ecologically friendly procedure. Cyclization of **2** cause the creation of new families of triazine and diazine derivatives. Moreover, the recently combined chemicals were verified as *anti-H. Pylori* activity. All tested samples have a bactericidal effect showing MBC/MIC Index for all tested samples ≤ 2. Conclusively, authors considered the molecular docking tentative study, for the synthesized compounds 7, 12 and 13. These compounds were proved to betalented candidates for supplementary studies to be used as an effective, efficient and safe *anti-H. pylori* medication. This study paves the way for this nanoparticle material as an as *anti-H. Pylori* therapy.

### Supplementary Information

Supplementary Information.
