# Supplementary material for: CuO nanoparticles for green synthesis of significant anti-Helicobacter pylori compounds with in silico studies
Source: Sci Rep. 2024 Jan 18;14:1608. doi: 10.1038/s41598-024-51708-1 (PMC10796945; doi:10.1038/s41598-024-51708-1)
Supplement: Supplementary file 1 — Supplementary Information. [file 41598_2024_51708_MOESM1_ESM.docx]

**Supporting Information**

**Spectral analysis**

**
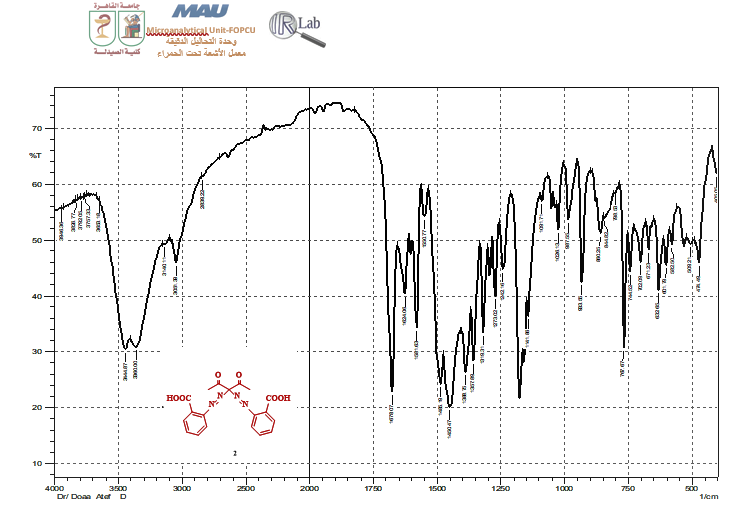
**

**Fig. 1: IR Spectrum of compound 2**

**
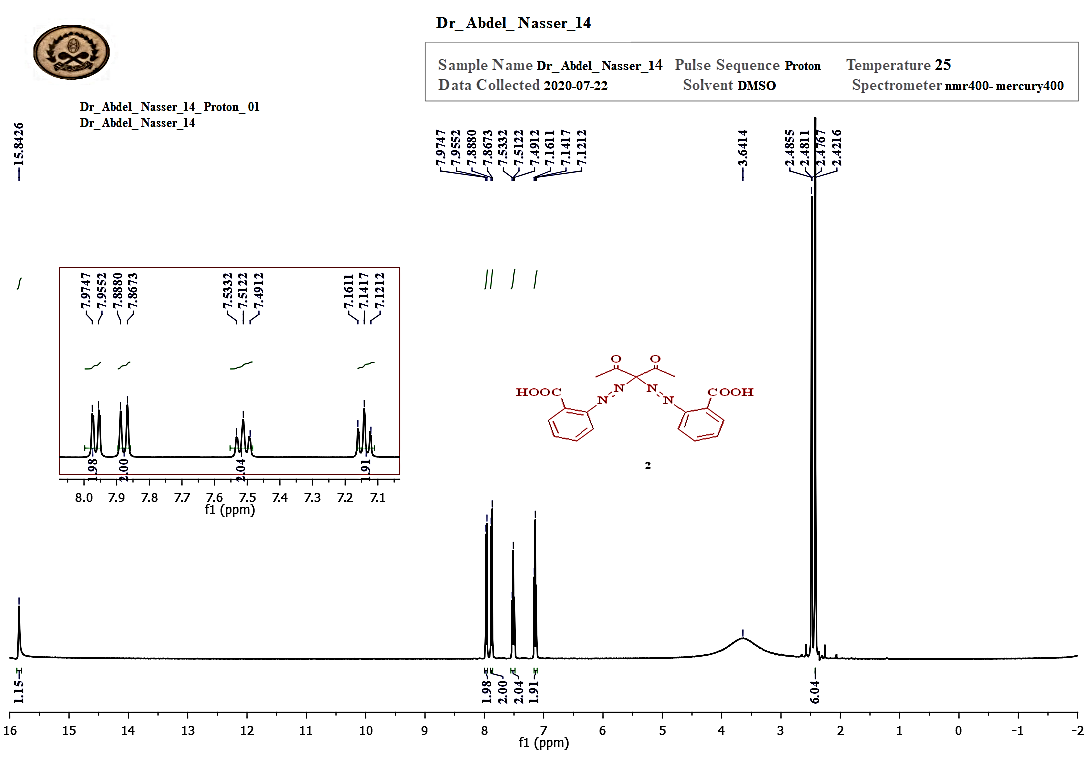
**

**Fig. 2: ^1^H-NMR Spectrum of compound 2**

**
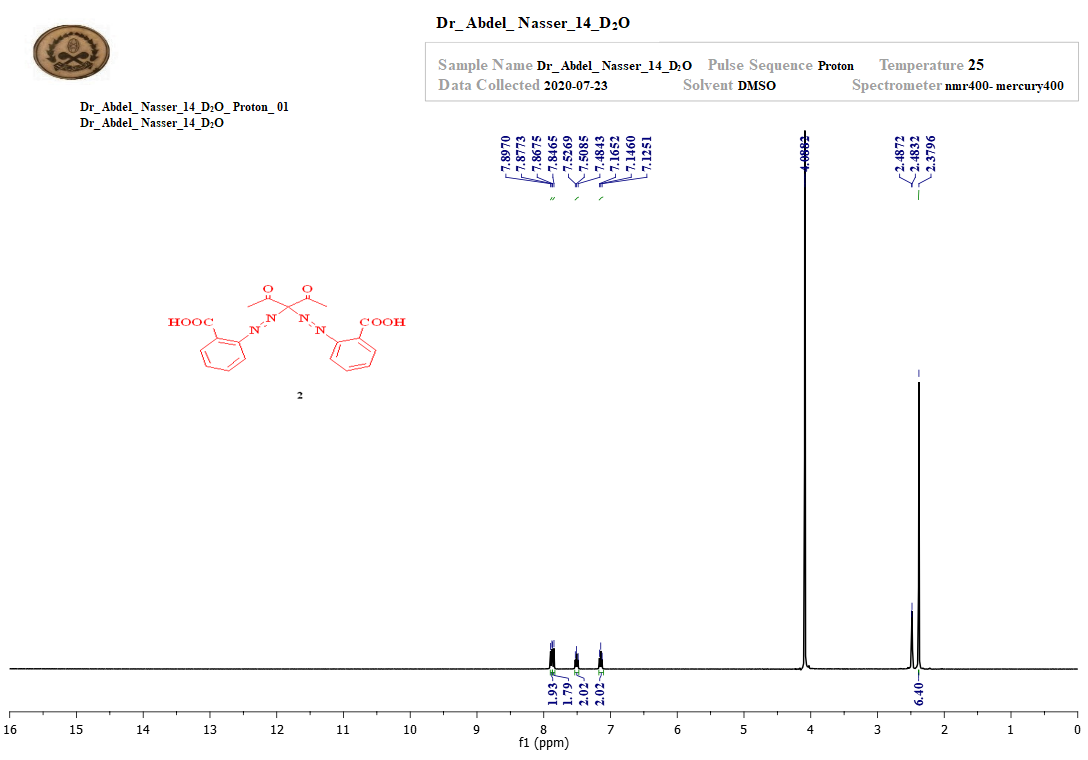
**

**Fig. 3: D_2_O Spectrum of compound 2**

**
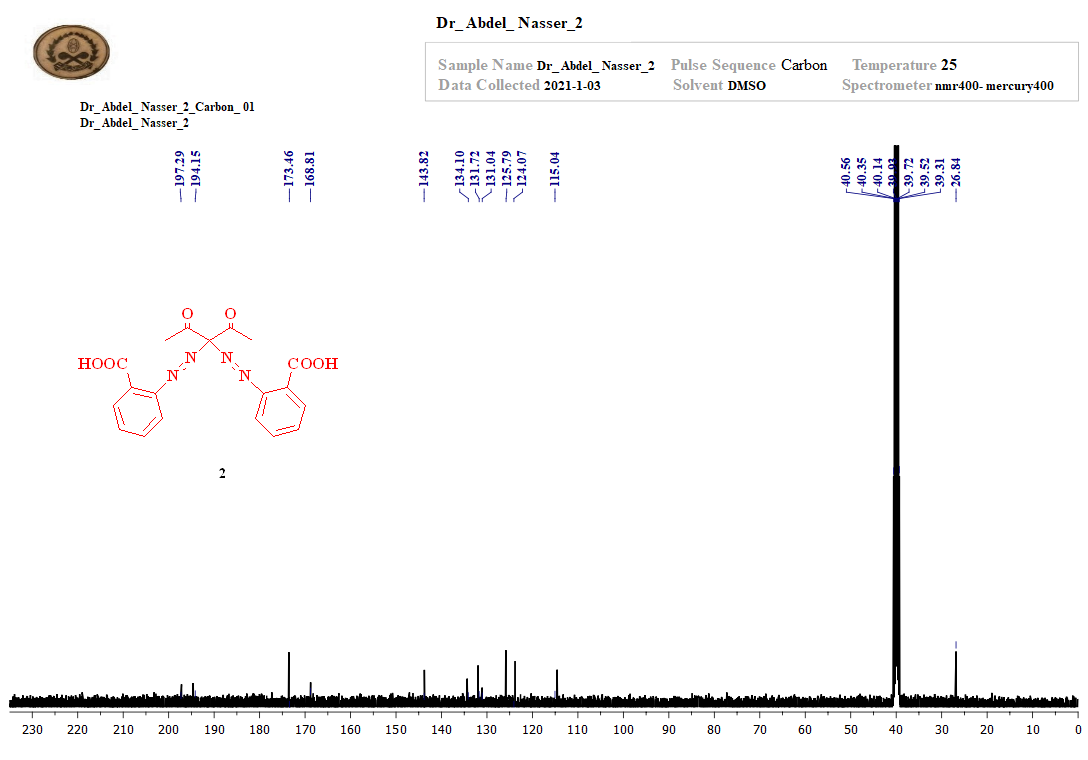
**

**Fig. 4: ^13^C-NMR Spectrum of compound 2**

**
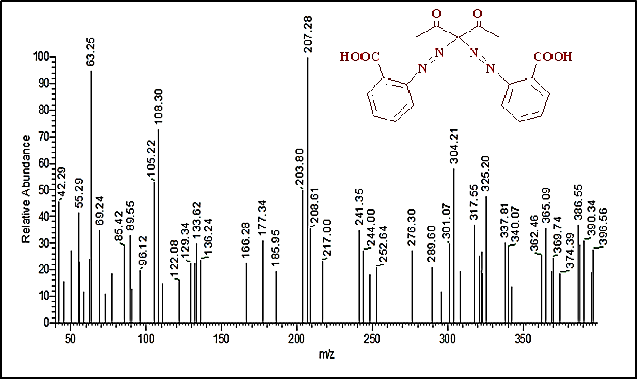
**

**Fig. 5: Mass spectra of compound 2**

**
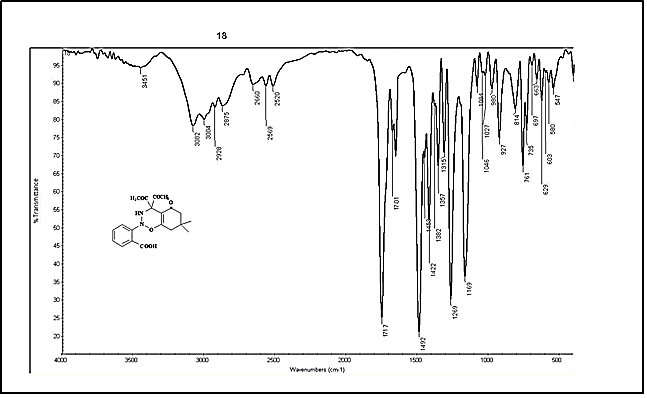
**

**Fig. 6: IR Spectrum of compound 3**


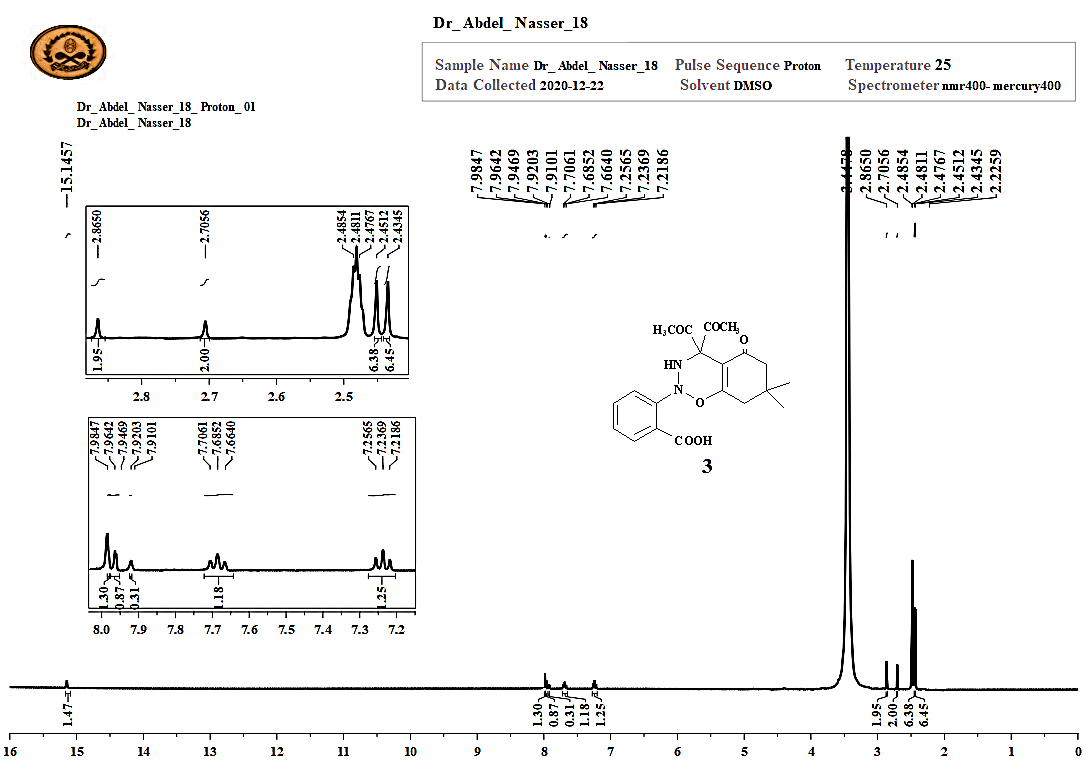


**Fig. 7: ^1^H-NMR Spectrum of compound 3**

**
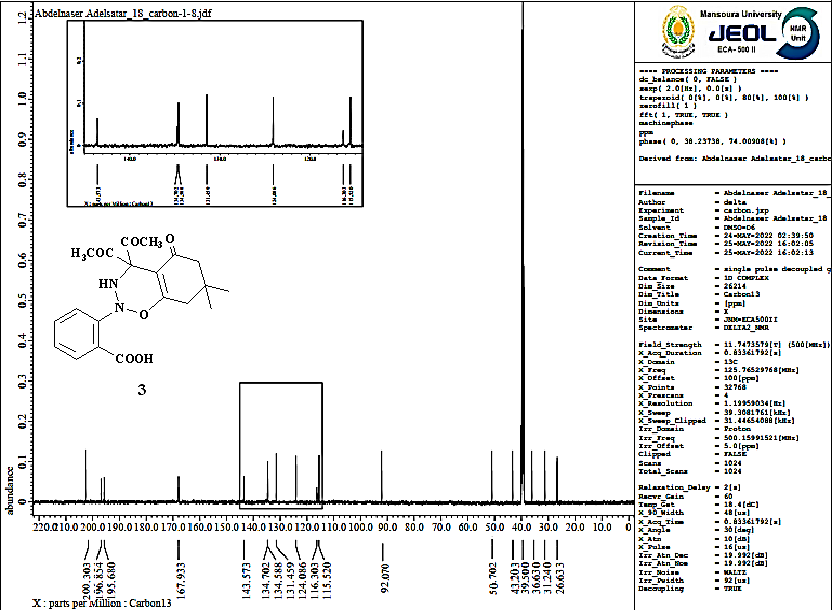
**

**Fig. 8: ^13^C-NMR Spectrum of compound 3**

**
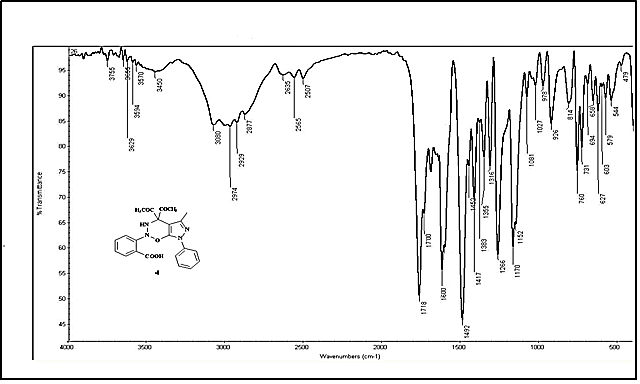
**

**Fig. 9: IR Spectrum of compound 4**

**
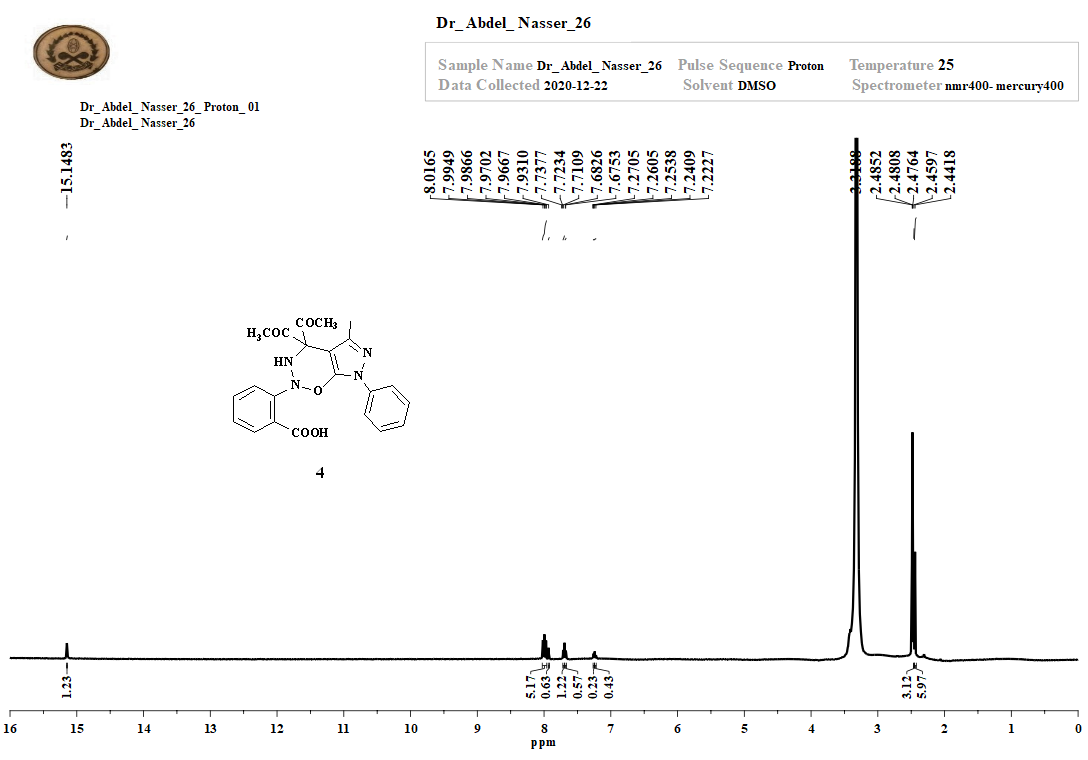
**

**Fig. 10: ^1^H-NMR Spectrum of compound 4**

**
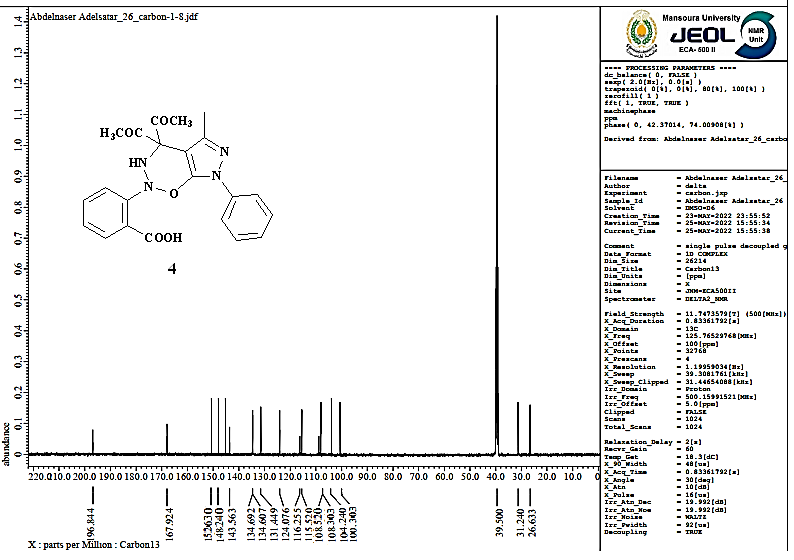
**

**Fig. 11: ^13^C-NMR Spectrum of compound 4**

**
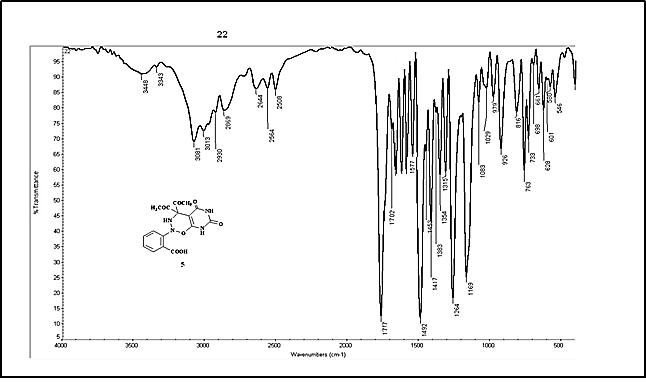
**

**Fig. 12: IR Spectrum of compound 5**

**
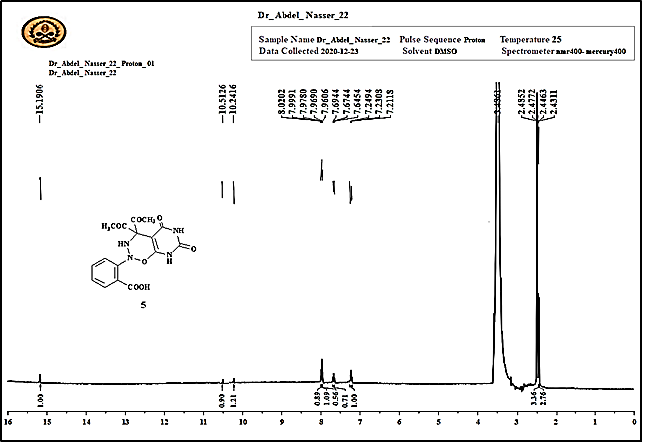
**

**Fig. 13: ^1^H-NMR Spectrum of compound**

**
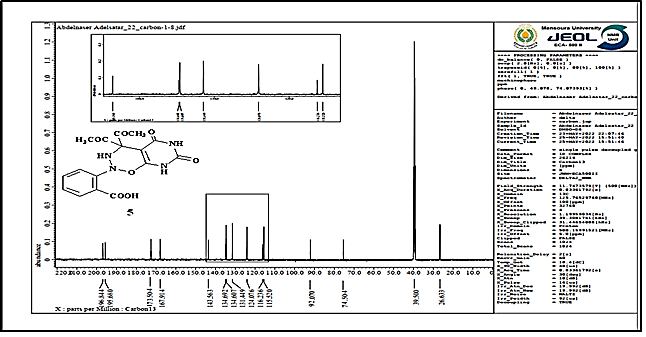
**

**Fig. 14: ^13^C-NMR Spectrum of compound 5**

**
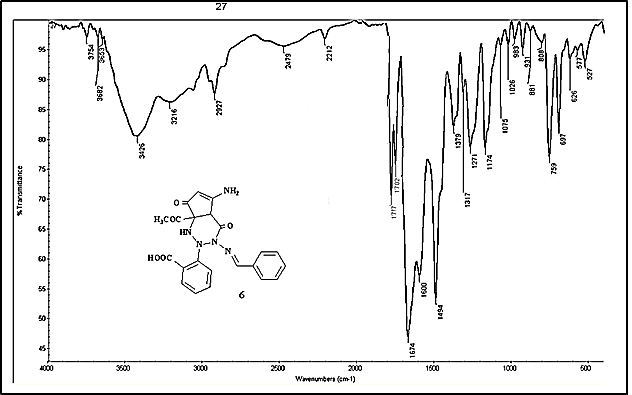
**

**Fig. 15: IR Spectrum of compound 6**

**
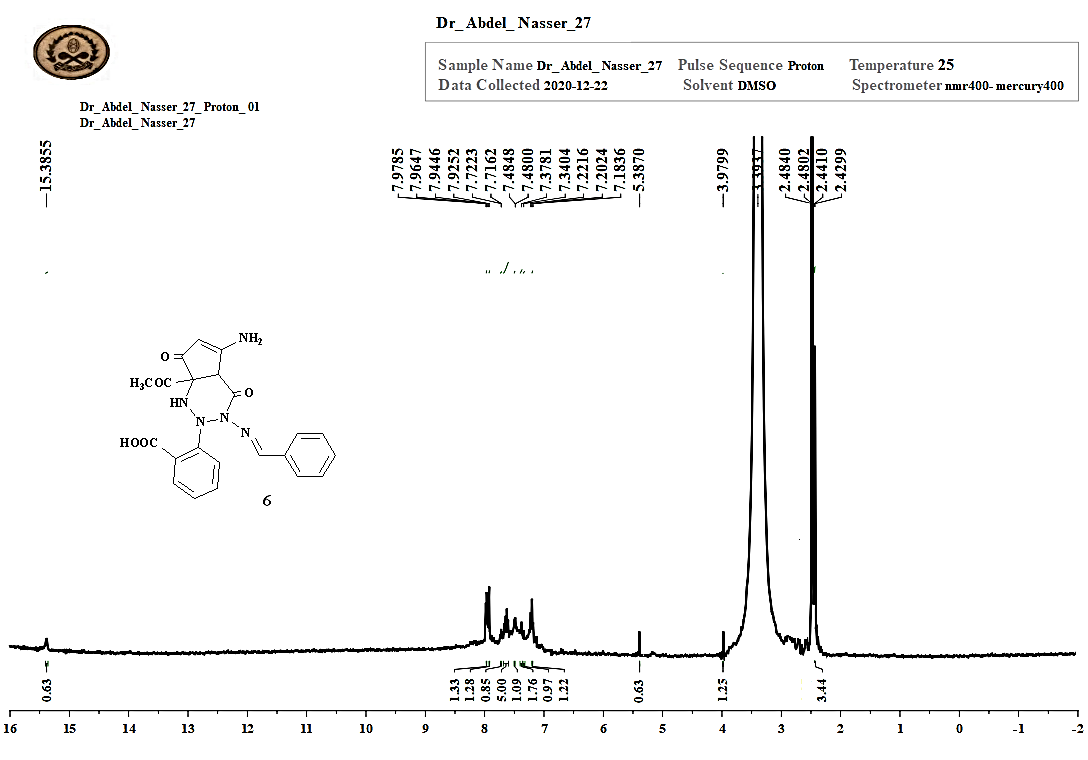
**

**Fig. 16: ^1^H-NMR Spectrum of compound 6**

**
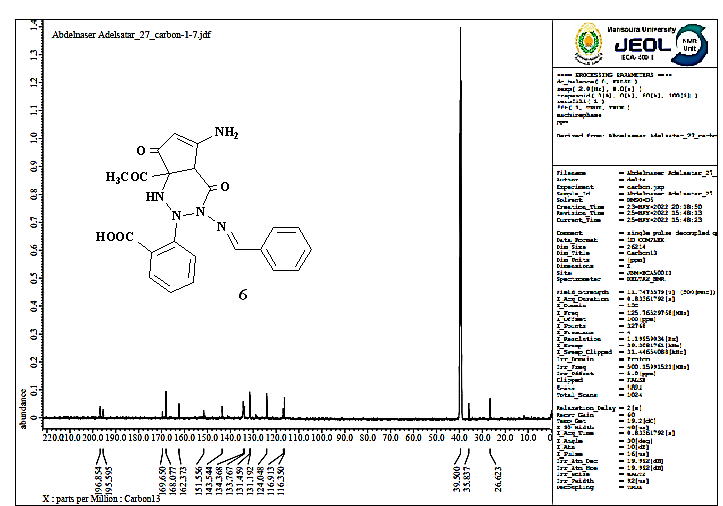
**

**Fig. 17: ^13^C-NMR Spectrum of compound 6**

**
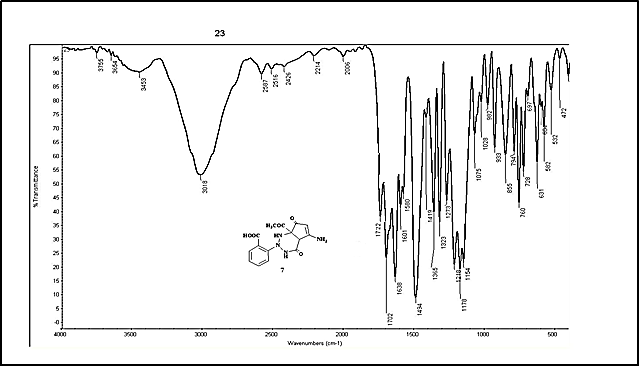
**

**Fig. 18: IR Spectrum of compound 7**

**
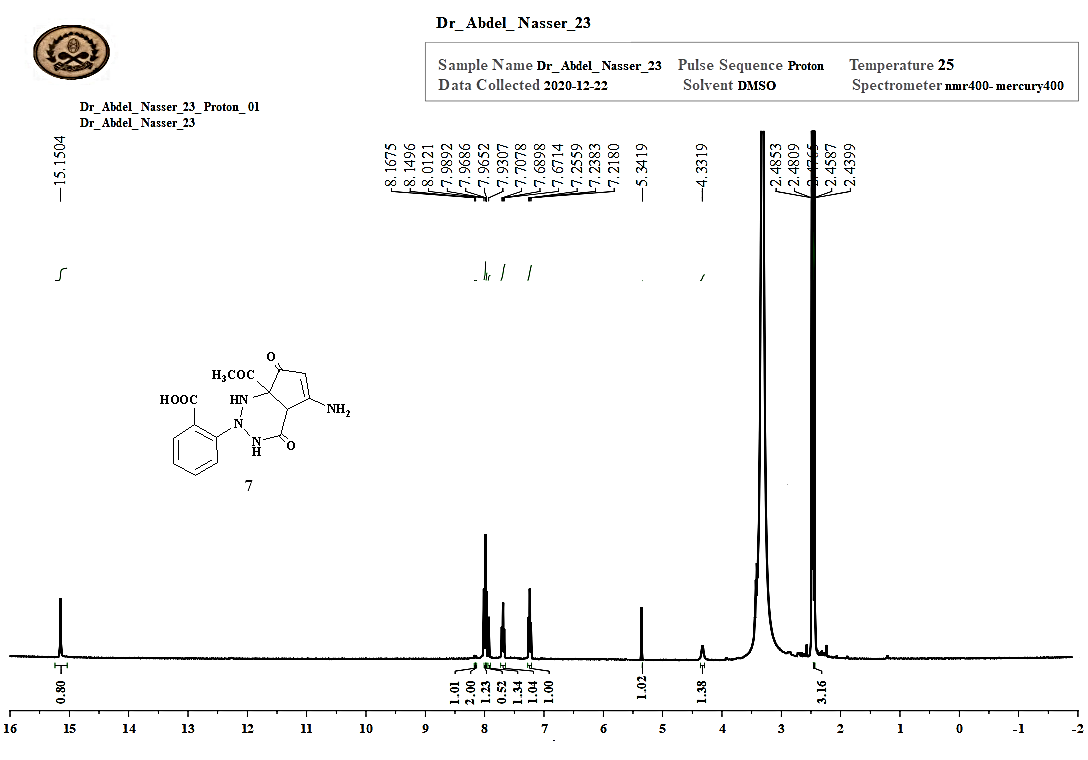
**

**Fig. 19: ^1^H-NMR Spectrum of compound 7**

**
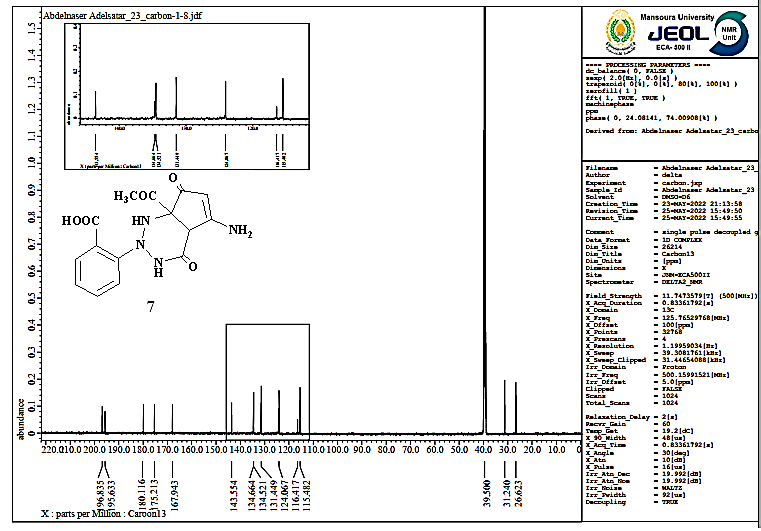
**

**Fig. 20: ^13^C-NMR Spectrum of compound 7**

**
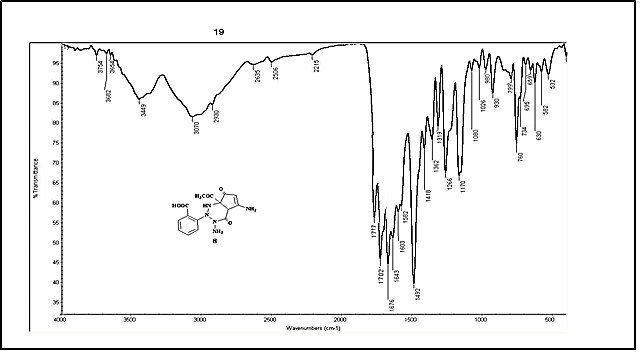
**

**Fig. 17: IR Spectrum of compound 8**

**
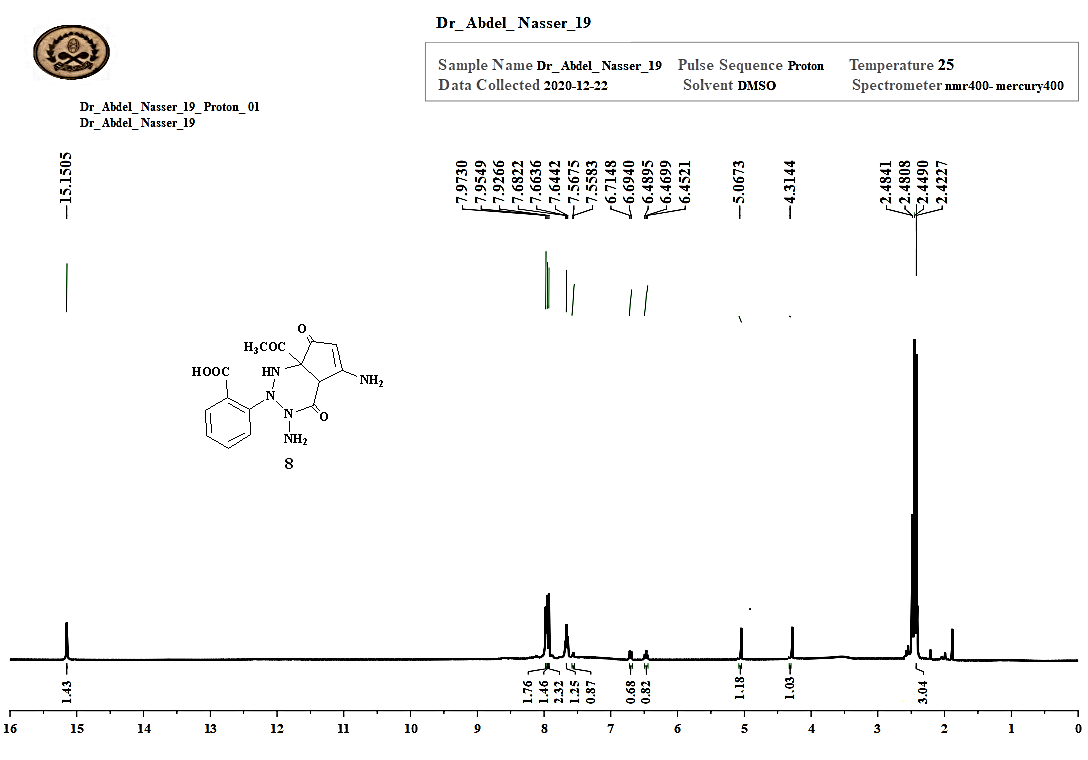
**

**Fig. 18: ^1^H-NMR Spectrum of compound 8**

**
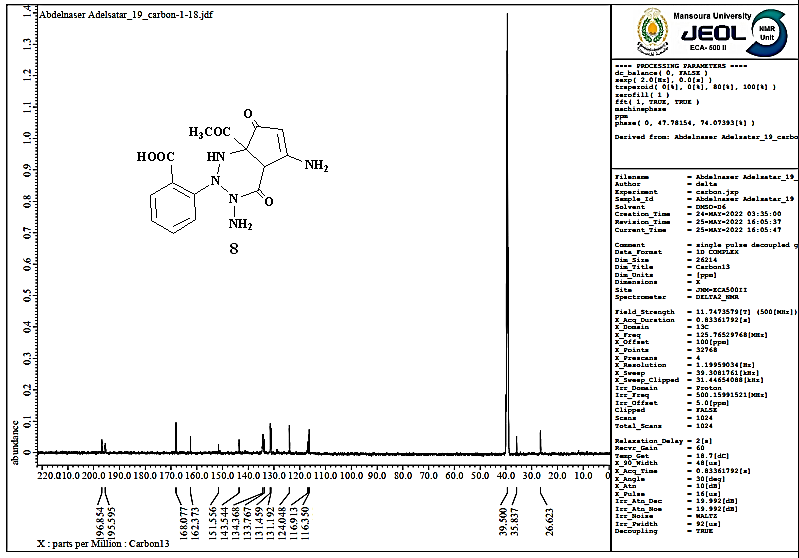
**

**Fig. 20: ^13^C-NMR Spectrum of compound 8**

**
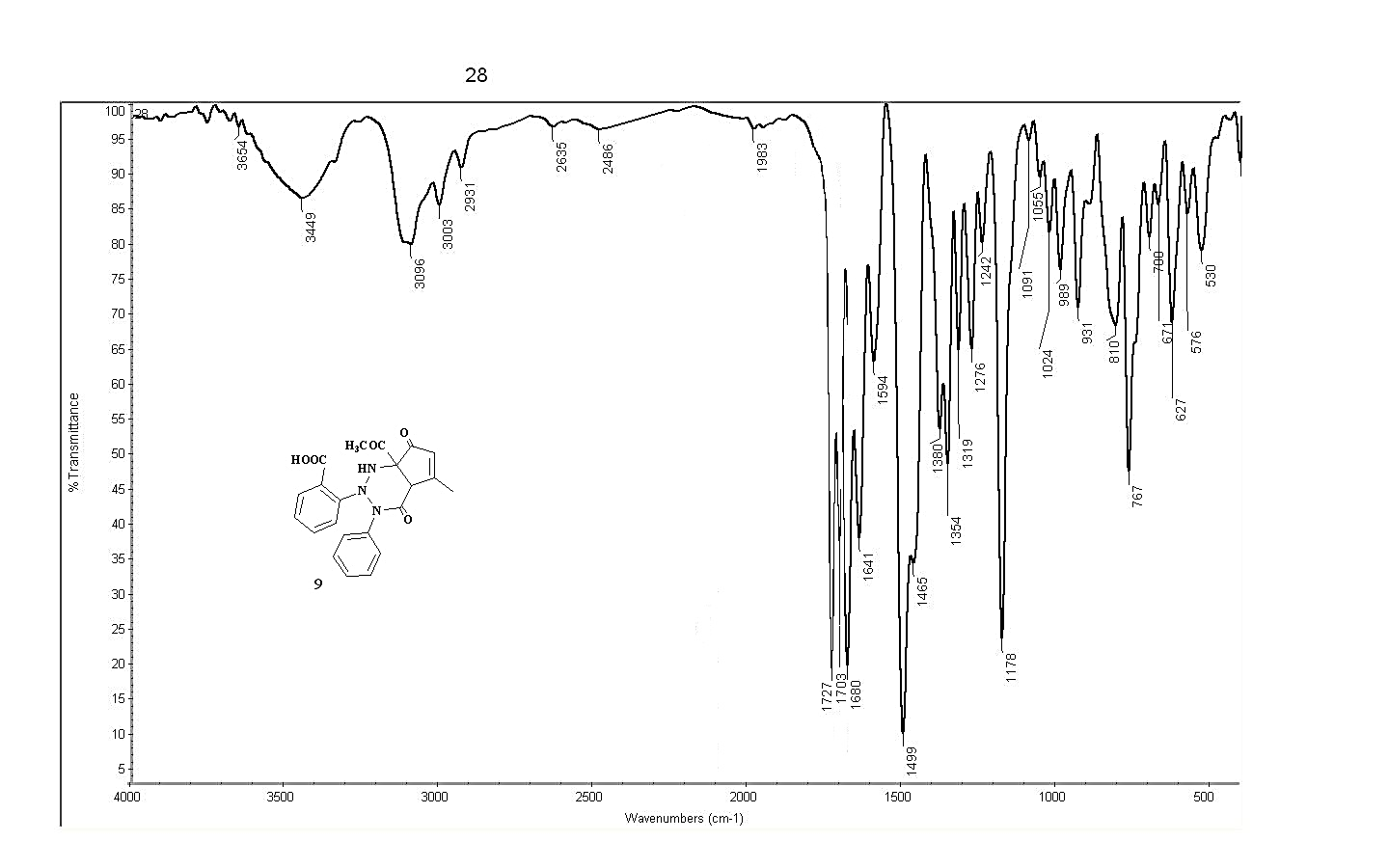
**

**Fig. 21: IR Spectrum of compound 9**

**
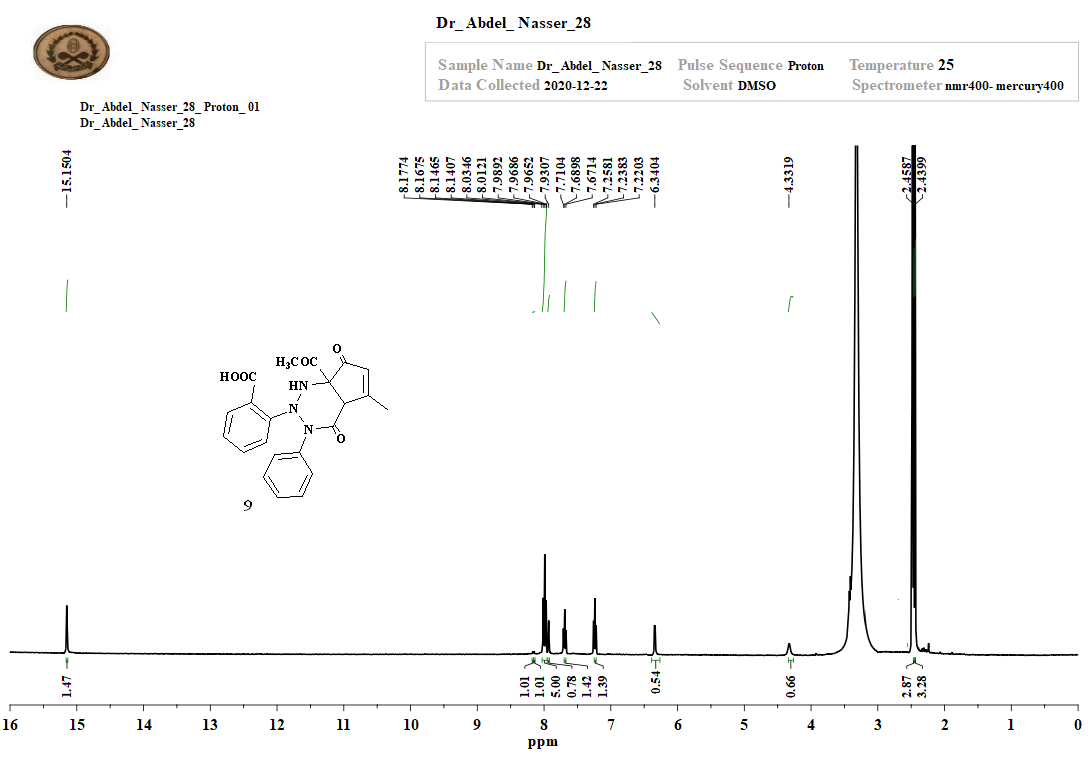
**

**Fig. 22: ^1^H-NMR Spectrum of compound 9**

**
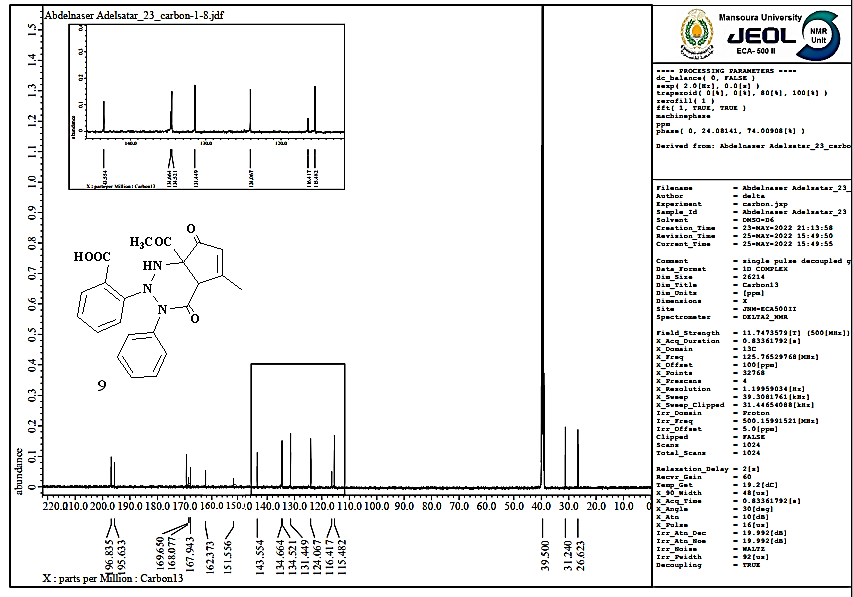
**

**Fig. 23: ^13^C-NMR Spectrum of compound 9**

**
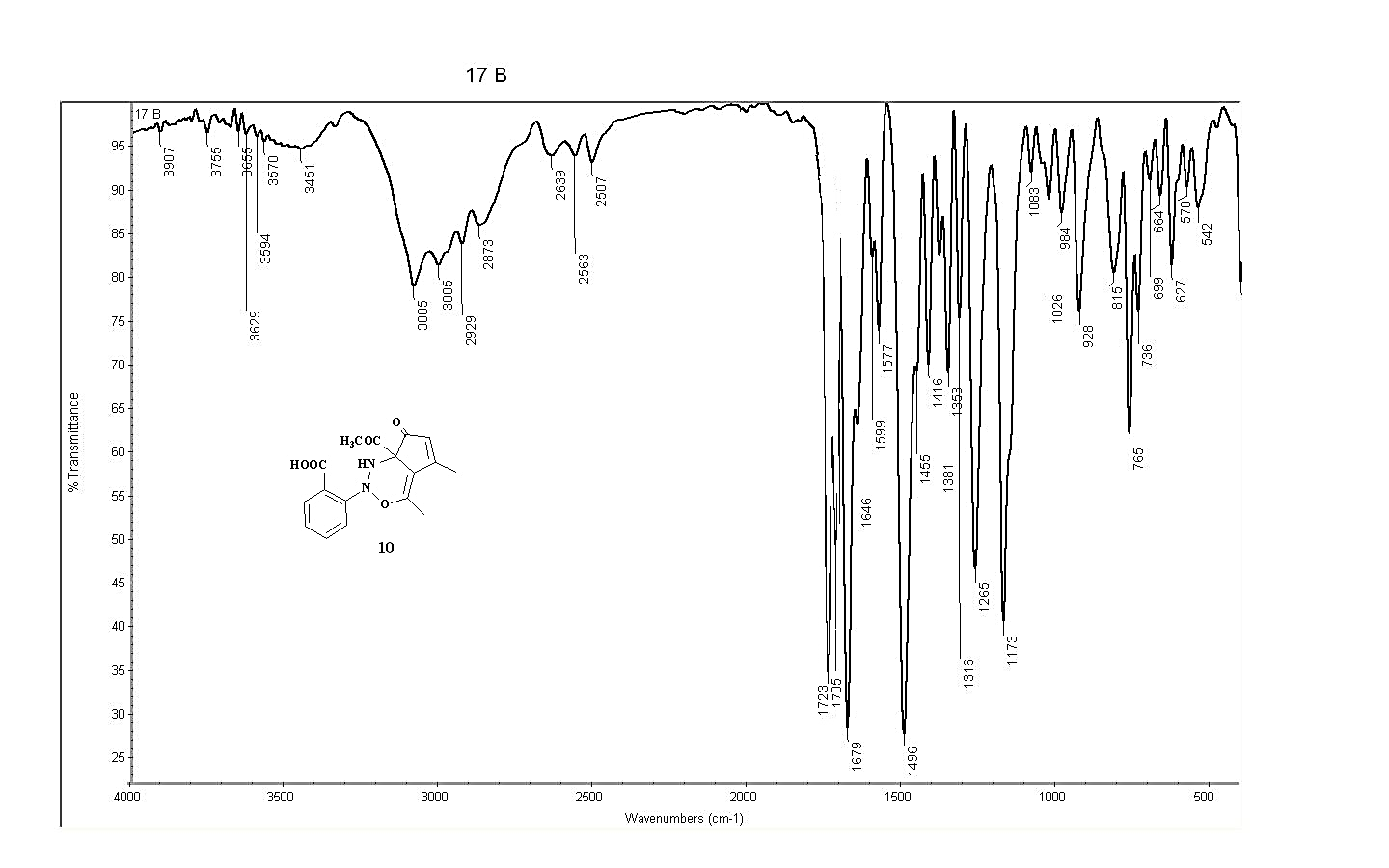
**

**Fig. 24: IR Spectrum of compound 10**

**
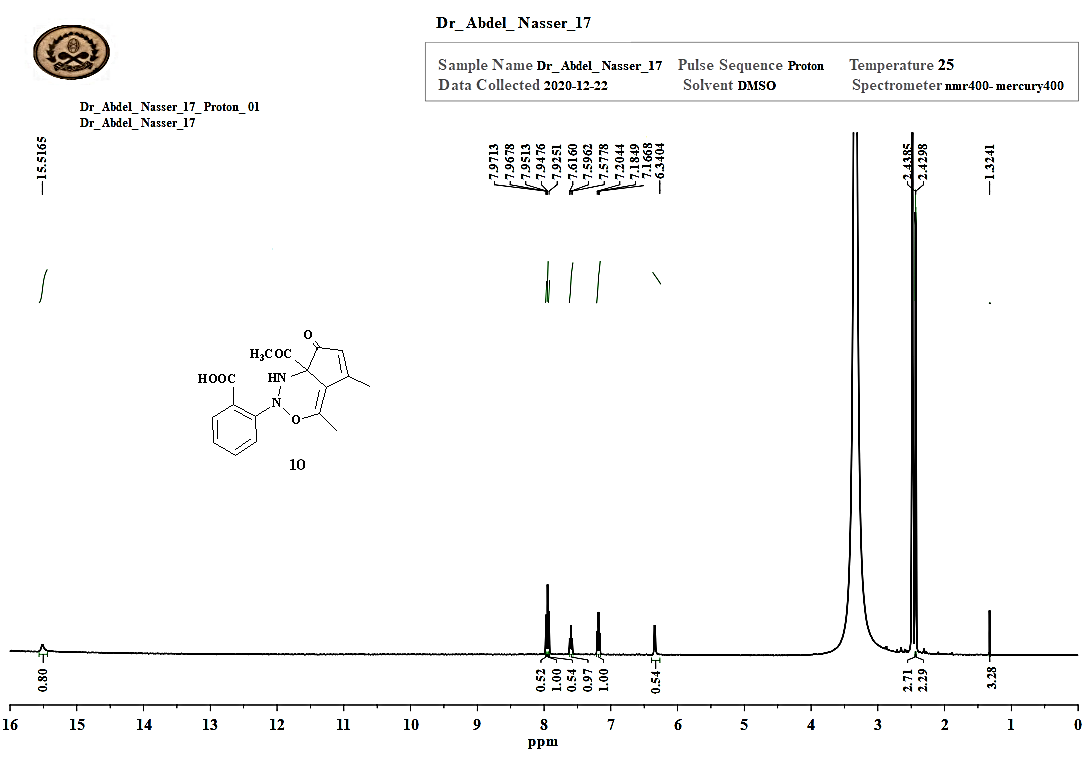
**

**Fig. 25: ^1^H-NMR Spectrum of compound 10**

**
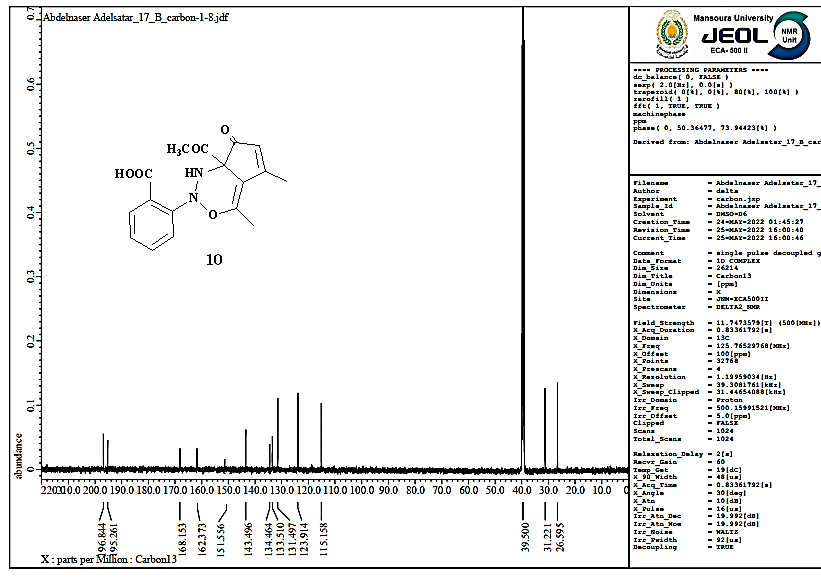
**

**Fig. 26: ^13^C-NMR Spectrum of compound 10**

**
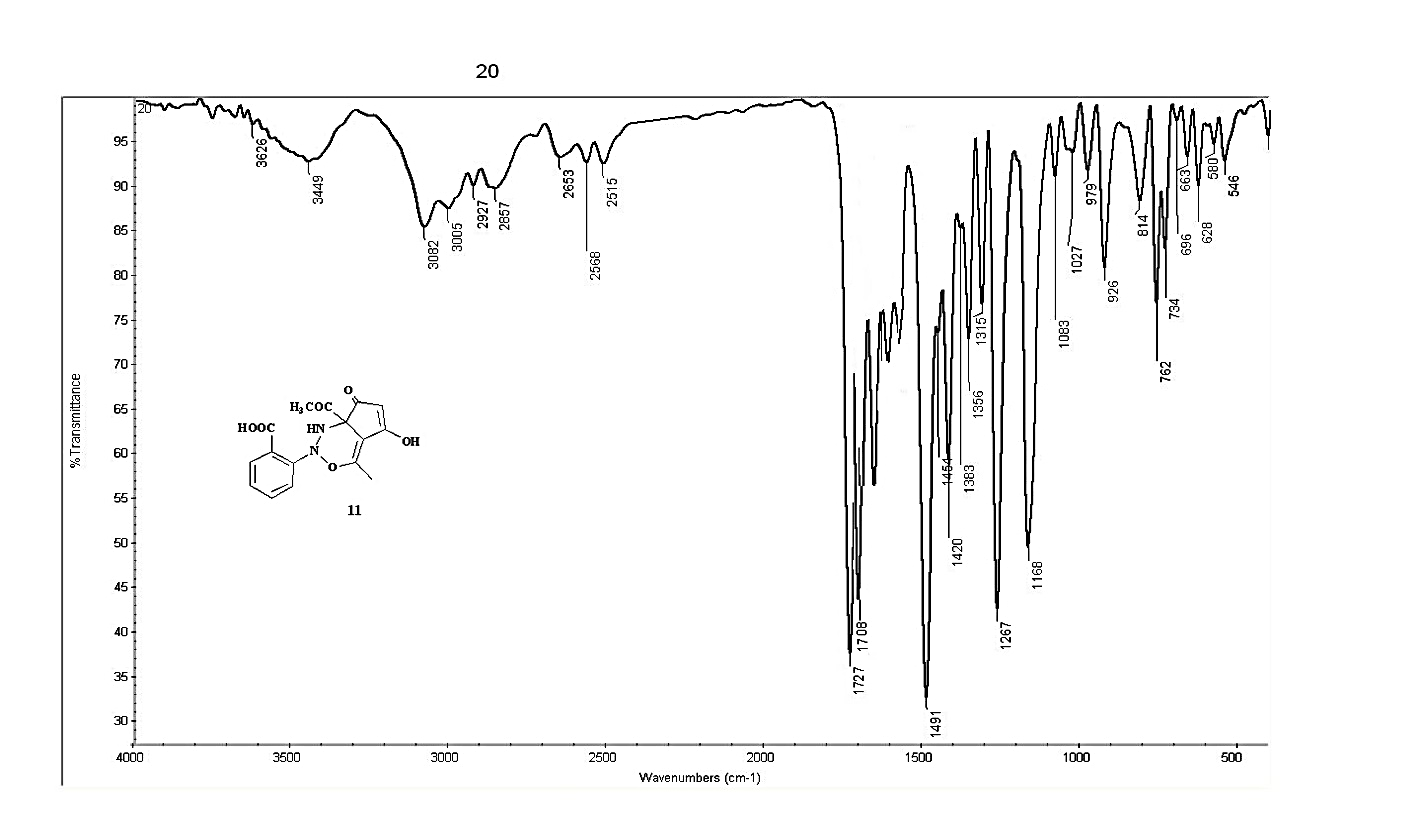
**

**Fig. 27: IR Spectrum of compound 11**

**
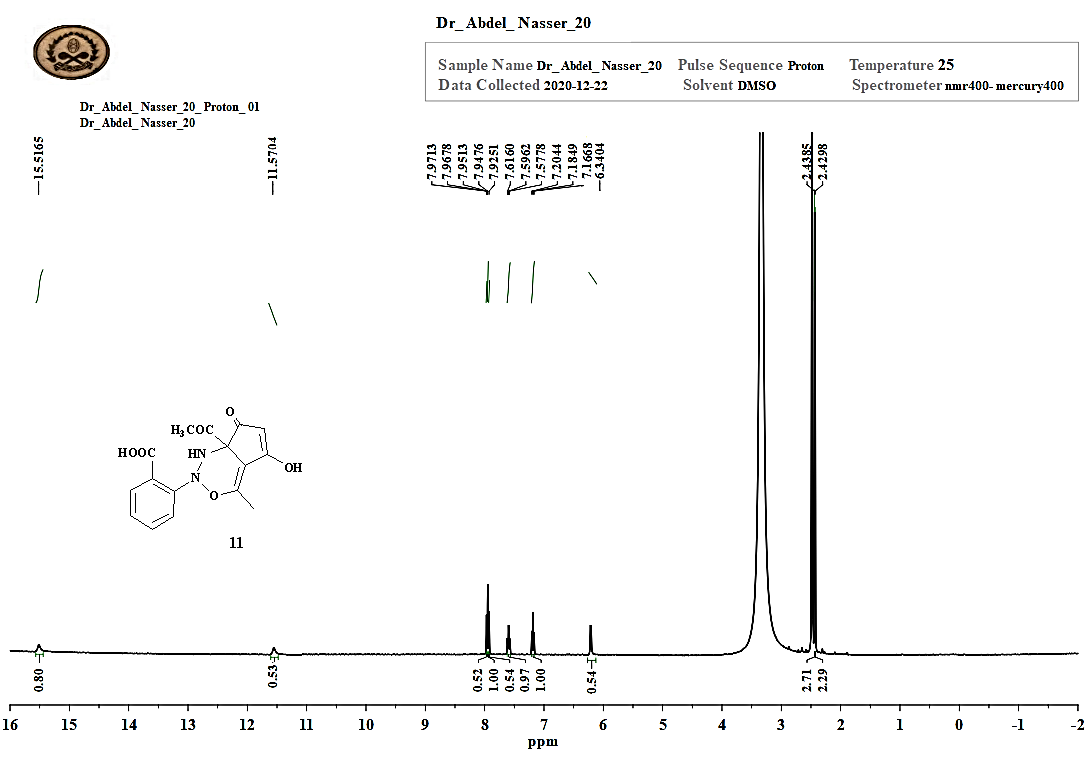
**

**Fig. 28: ^1^H-NMR Spectrum of compound 11**

**
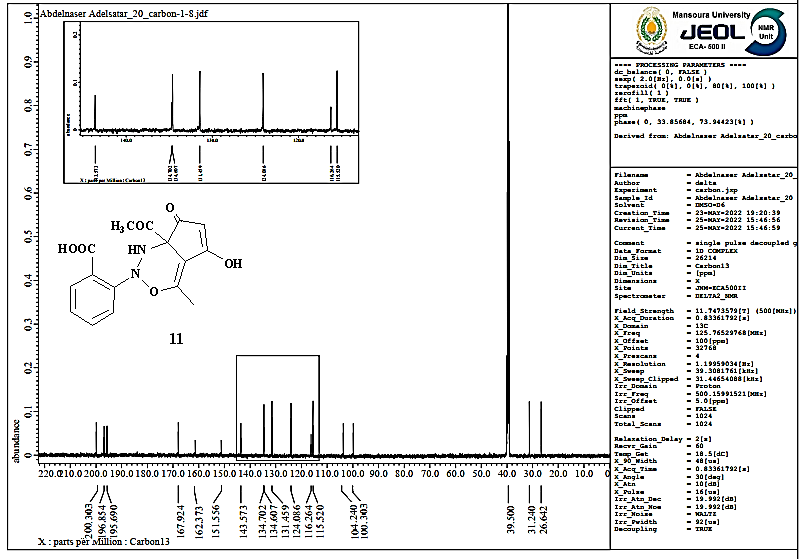
**

**Fig. 29: ^13^C-NMR Spectrum of compound 11**

**
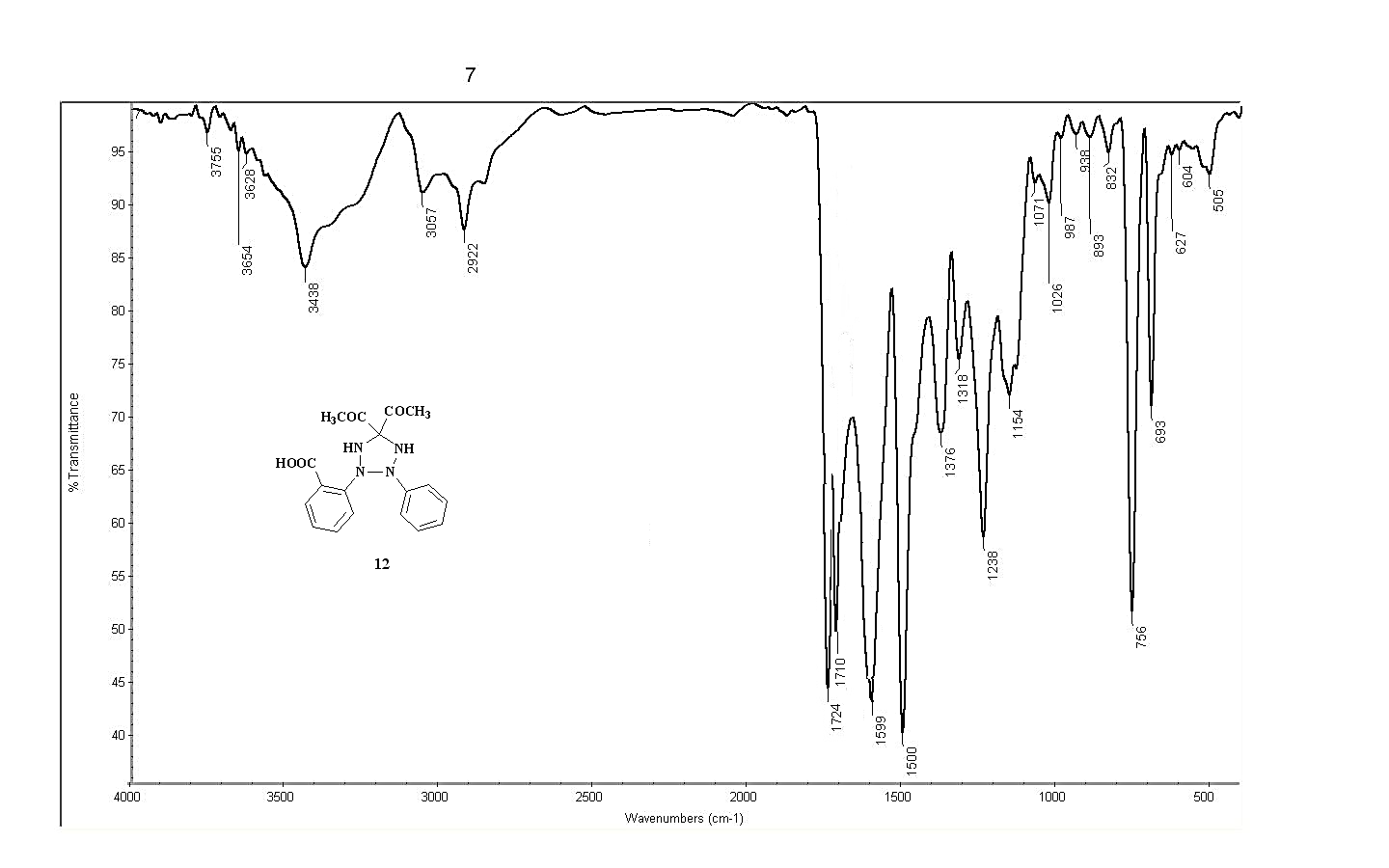
**

**Fig. 30: IR Spectrum of compound 12**

**
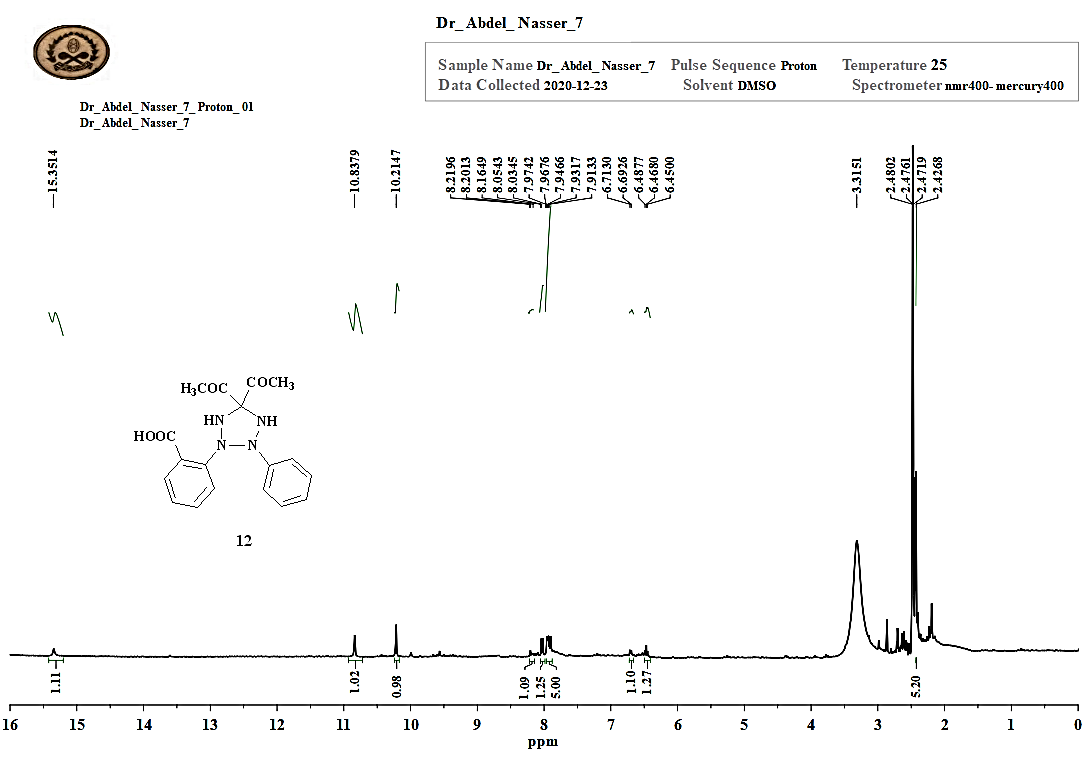
**

**Fig. 31: ^1^H-NMR Spectrum of compound 12**

**
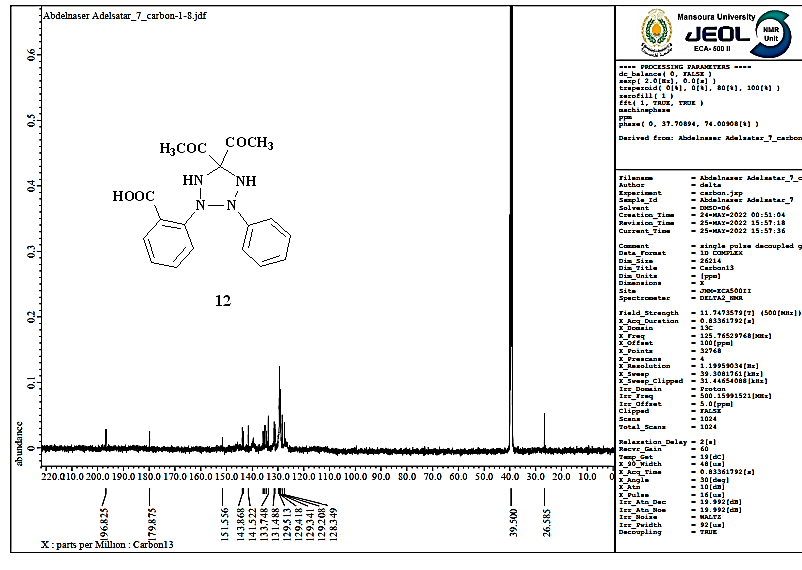
**

**Fig. 32: ^13^C-NMR Spectrum of compound 12**

**
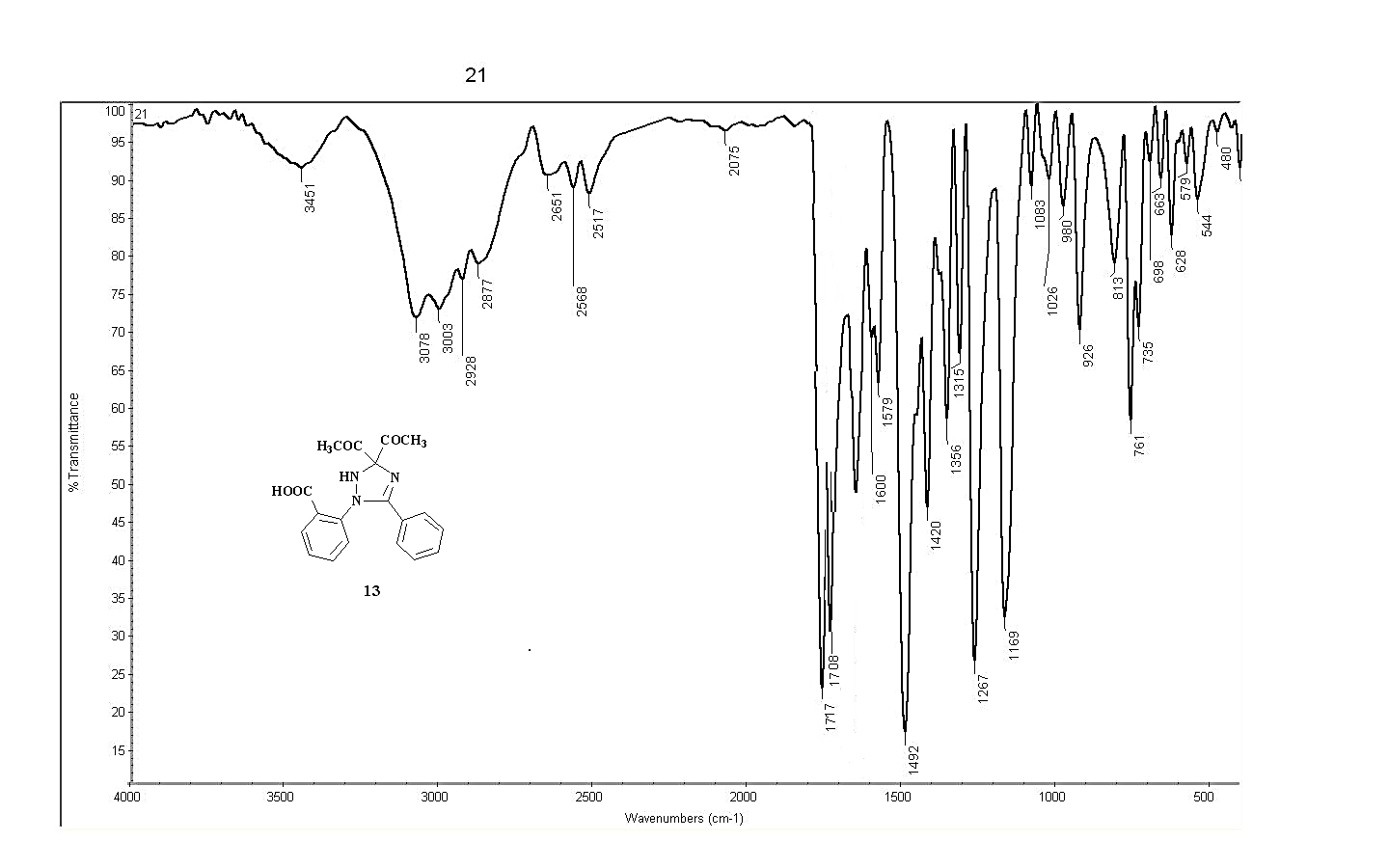
**

**Fig. 33: IR Spectrum of compound 13**

**
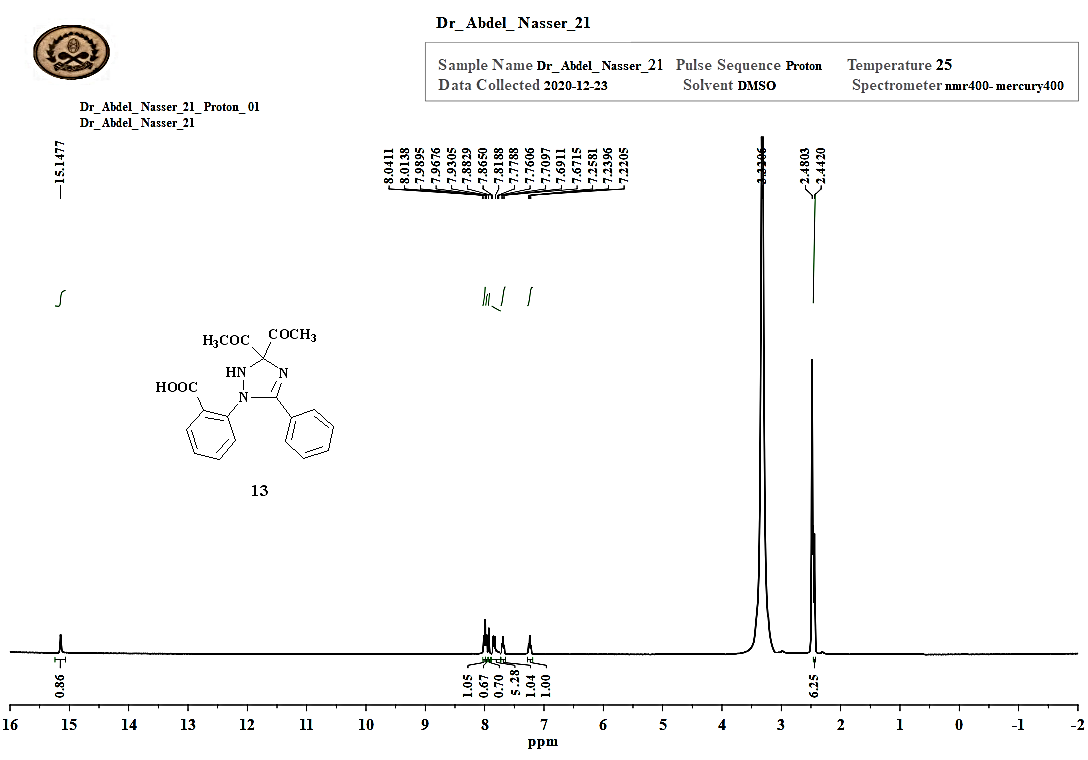
**

**Fig. 34: ^1^H-NMR Spectrum of compound 13**

**
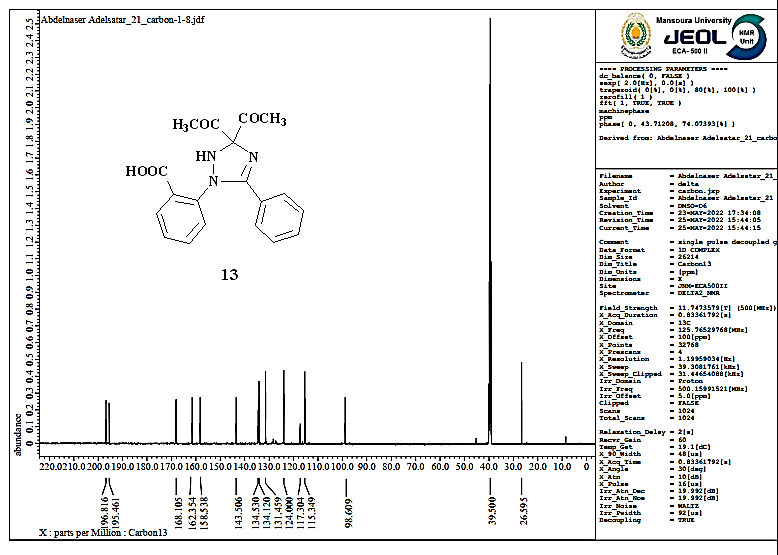
**

**Fig. 35: ^13^C-NMR Spectrum of compound 13**

**
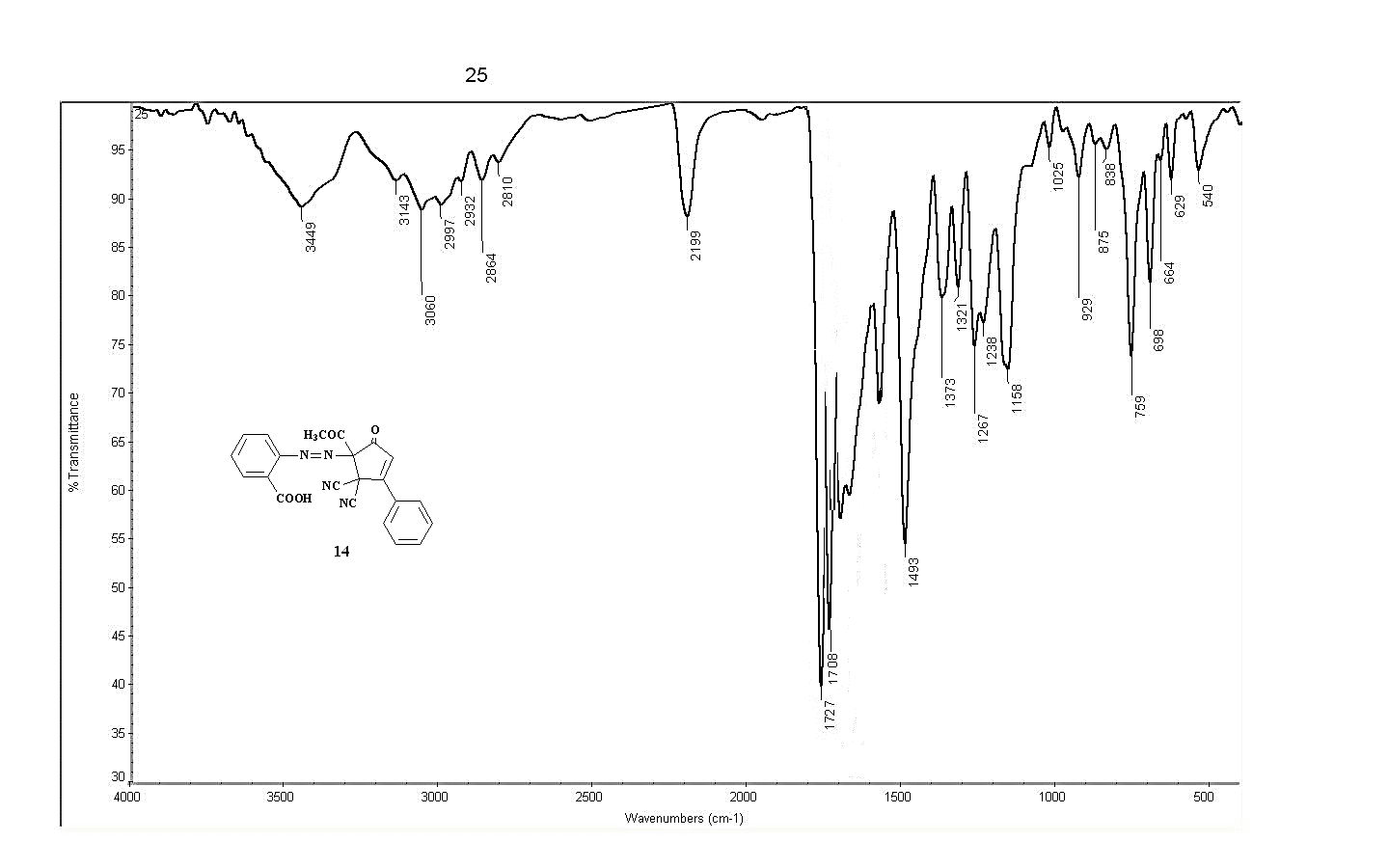
**

**Fig. 36: IR Spectrum of compound 14**

**
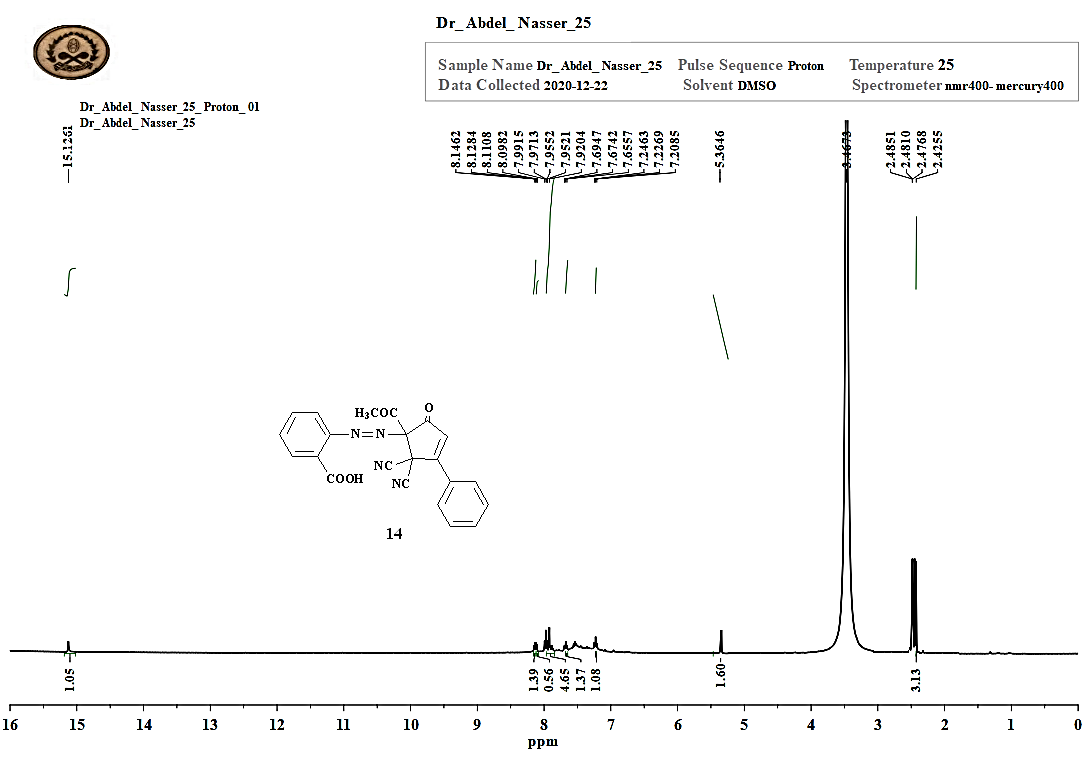
**

**Fig. 37: ^1^H-NMR Spectrum of compound 14**

**
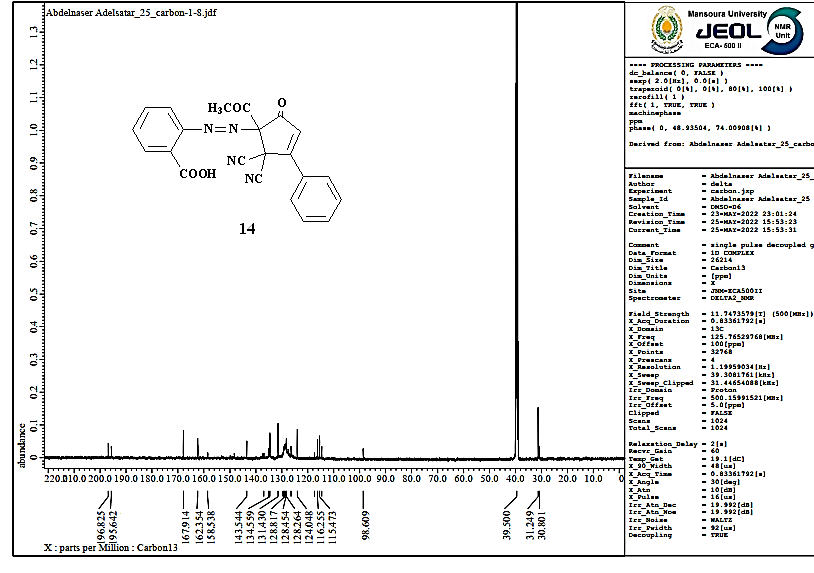
**

**Fig. 38: ^13^C-NMR Spectrum of compound 14**

**
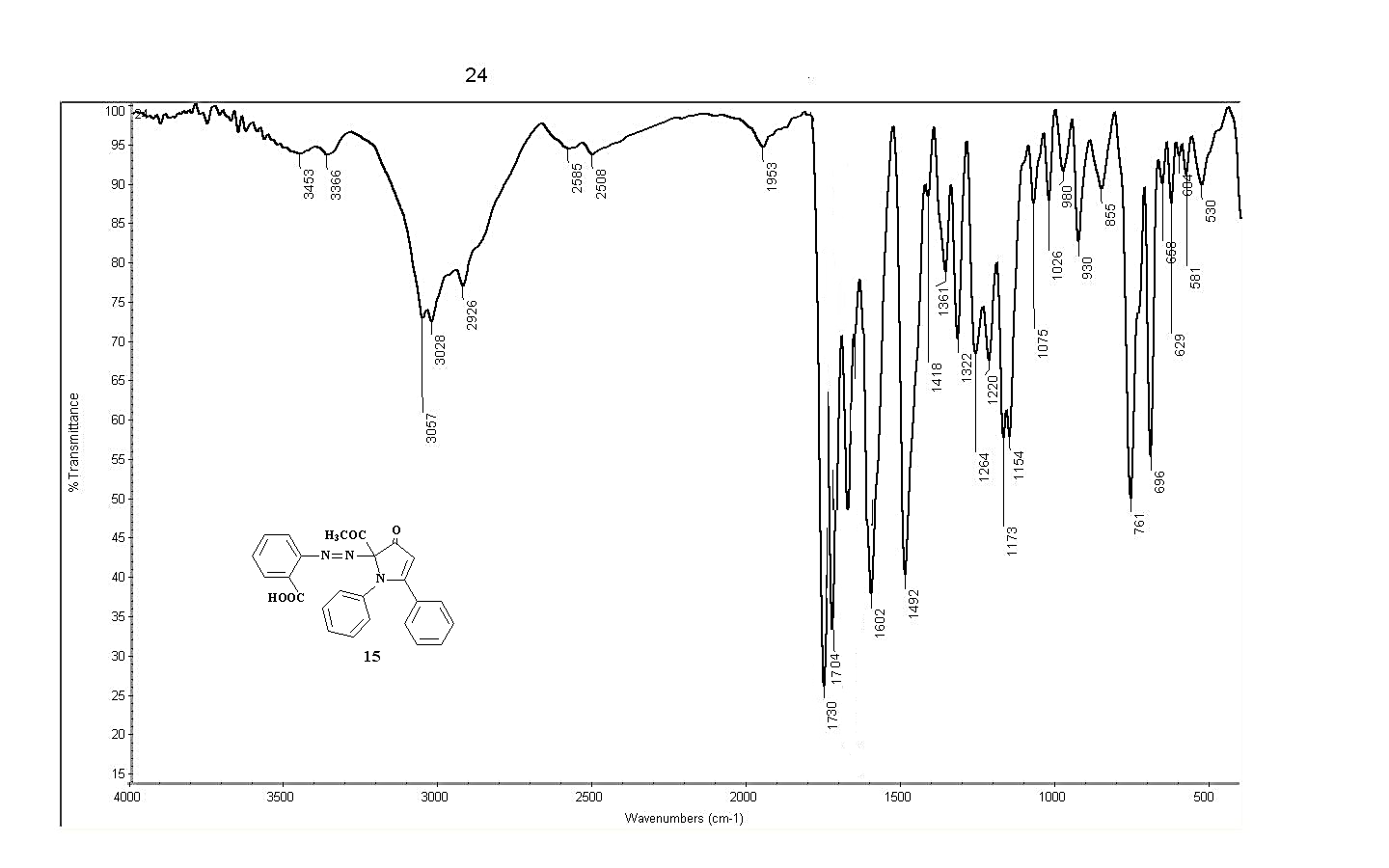
**

**Fig. 39: IR Spectrum of compound 15**

**
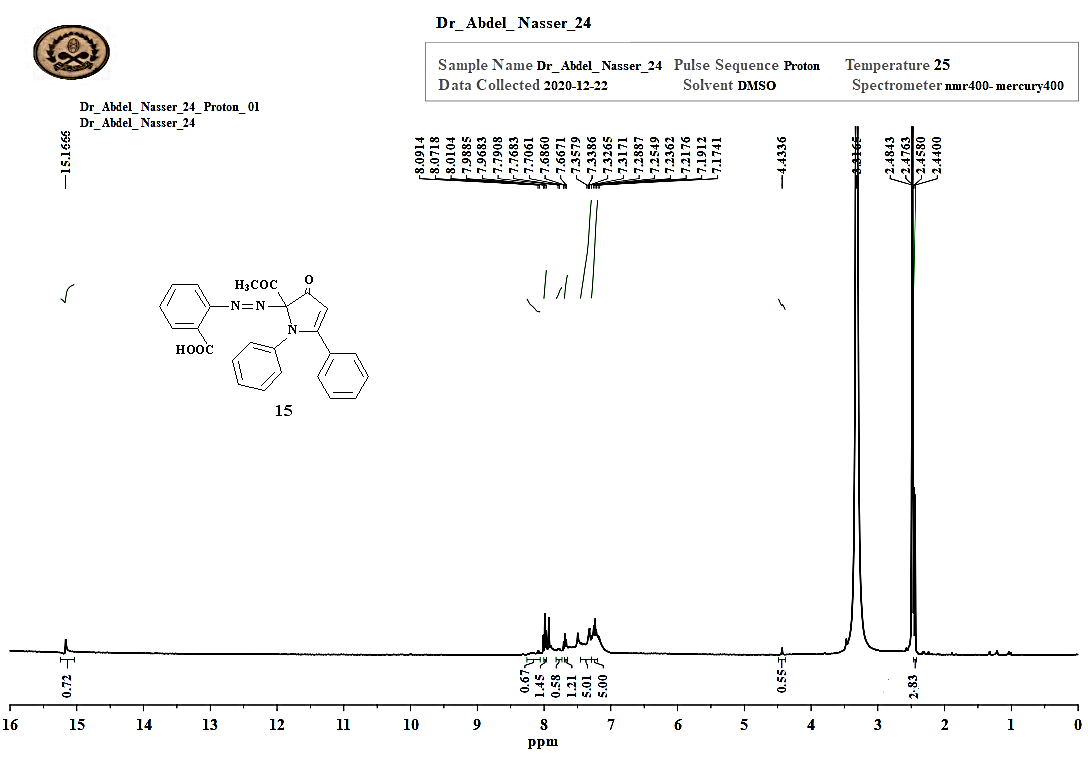
**

**Fig. 40: ^1^H-NMR Spectrum of compound 15**
